# Topological Analysis of Hedgehog Acyltransferase, a Multipalmitoylated Transmembrane Protein*[Fn FN2]

**DOI:** 10.1074/jbc.M114.614578

**Published:** 2014-12-12

**Authors:** Antonio D. Konitsiotis, Biljana Jovanović, Paulina Ciepla, Martin Spitaler, Thomas Lanyon-Hogg, Edward W. Tate, Anthony I. Magee

**Affiliations:** From the ‡ Molecular Medicine Section and; ‖ FILM (Facility for Imaging by Light Microscopy), National Heart and Lung Institute,; § Department of Chemistry, and; ¶ Institute of Chemical Biology Imperial College London, Sir Alexander Fleming Building, South Kensington, London SW7 2AZ, United Kingdom

**Keywords:** Cancer, Click Chemistry, Hedgehog Signaling Pathway, Protein Palmitoylation, Transmembrane Domain, HHAT, MBOAT, Acyltransferase, Topology

## Abstract

Hedgehog proteins are secreted morphogens that play critical roles in development and disease. During maturation of the proteins through the secretory pathway, they are modified by the addition of N-terminal palmitic acid and C-terminal cholesterol moieties, both of which are critical for their correct function and localization. Hedgehog acyltransferase (HHAT) is the enzyme in the endoplasmic reticulum that palmitoylates Hedgehog proteins, is a member of a small subfamily of membrane-bound *O-*acyltransferase proteins that acylate secreted proteins, and is an important drug target in cancer. However, little is known about HHAT structure and mode of function. We show that HHAT is comprised of ten transmembrane domains and two reentrant loops with the critical His and Asp residues on opposite sides of the endoplasmic reticulum membrane. We further show that HHAT is palmitoylated on multiple cytosolic cysteines that maintain protein structure within the membrane. Finally, we provide evidence that mutation of the conserved His residue in the hypothesized catalytic domain results in a complete loss of HHAT palmitoylation, providing novel insights into how the protein may function *in vivo*.

## Teaser

**Background:** Hedgehog acyltransferase (HHAT) palmitoylates hedgehog proteins and is a potential target in cancer.

**Results:** HHAT has ten transmembrane domains, two reentrant loops, and four palmitoylation sites.

**Conclusion:** HHAT topology is determined, and protein is multipalmitoylated, which modulates protein function.

**Significance:** Elucidating HHAT topology and posttranslational modifications is crucial to understand its acyltransferase activity and to develop new strategies to treat cancer.

## Introduction

Hedgehog (Hh)^7^ family proteins are secreted morphogens that play a significant role during embryonic development in determining organogenesis and anterior-posterior patterning of various tissues, including the central nervous system and limb and digit formation (1, 2). Aberrant activation of Hh signaling in various cancers results in promotion of cancer growth and metastasis (3, 4), and small molecule inhibitors for this pathway are in use in various clinical trials.

A unique feature of the Hh proteins is that, during maturation through the secretory pathway, they are posttranslationally modified by the addition of a cholesterol moiety to the C terminus via an ester linkage, whereas a palmitate (C16:0) fatty acid is also added on the conserved N-terminal cysteine residue via an amide linkage (5). Palmitoylation of Hh proteins is catalyzed by the protein acyltransferase (PAT) Hedgehog acyltransferase (HHAT) (6).

These modifications produce the mature and functional Hh signaling molecule and are crucial for the correct function of the protein. They not only direct the formation of large Hh multimers upon secretion from the producing cells, but they also determine the proper release and targeting of the proteins and have a significant effect on the potency of the protein to activate the signaling pathway on the receiving cells (7–9). For these reasons, disrupting the posttranslational modification of Hh proteins and, hence, inhibiting the formation of functional signaling molecules is an attractive new method of inhibiting the Hh pathway in cancer.

HHAT is a multipass transmembrane (TM) domain protein located in the endoplasmic reticulum (ER) of cells (6, 10) and a member of a small subgroup of the membrane-bound *O-*acyltransferase (MBOAT) superfamily of proteins that specifically acylates secreted proteins. Other members of this important subgroup include Porcupine (PORCN), which palmitoyleoylates (C16:1) the Wnt family of proteins, and ghrelin-*O*-acyltransferase (GOAT), which octanoylates (C8:0) the appetite-sensing peptide ghrelin (11). All MBOAT proteins are characterized by their MBOAT homology domain, a region of highly conserved residues including an invariant His residue (His-379 in the case of HHAT) and a highly conserved Asn or, in the case of HHAT, Asp (Asp-339) residue 30–45 amino acids upstream (11, 12). These amino acids are proposed to be catalytic, although this is still not clear, especially for HHAT, where mutation of the His to an Ala still retains significant PAT activity (13). Recent studies by our group and others provided proof of principle that inhibiting HHAT function is a valid method of inhibiting Hh signaling in cancer (14–16).

Despite the importance of HHAT in Hh signaling and its therapeutic potential in cancer, little is understood about the structure of the protein and the identity of the catalytically important amino acids, information that may guide future studies in the development of small molecule inhibitors of HHAT and into other functions HHAT may have in the cell. In this study, we determine HHAT topology using a variety of experimental methods. Our data suggest that HHAT contains ten TM domains and two reentrant loops (RLs) and that the invariant His-379 is luminal, whereas Asp-339 is on the cytosolic side of the ER. Furthermore, we show that HHAT is itself palmitoylated at multiple cytoplasmic Cys residues and that His-379 is critical for the palmitoylation of HHAT, whereas a conserved Cys-324 appears to significantly modulate protein topology.

## EXPERIMENTAL PROCEDURES

### 

#### 

##### Cell Culture and Transfection

HEK293a and HeLa cells were cultured in high-glucose (4.5 g/liter) DMEM supplemented with Glutamax (Invitrogen) containing 10% FBS (Sigma). All cells were grown at 37 °C in a humidified incubator under 5% CO_2_. Cells were transfected at 70% confluence using Turbofect (Thermo Fisher Scientific, Cramlington, UK) according to the specifications of the manufacturer.

##### Plasmid Construction and Mutagenesis

The full-length human HHAT cDNA (accession no. BC117130) expression vector with C-terminal V5 and His_6_ epitopes has been described previously (15). The cloned cDNA sequence carries a missense mutation compared with the human *HHAT* consensus sequence, a serine-to-aspartate change in position 182 of the protein. Prior to proceeding with making mutants for HHAT for topology analysis, the missense mutation was corrected to serine by QuikChange II site-directed mutagenesis (forward primer, CTACTACACCAGCTTCAGCCTGGAGCTCTGCTGGCAGCAGC; reverse primer, CAGGCTGAAGCTGGTGTAGTAGAGGCAGCGAACGGTCAGCG). All subsequent HHAT mutants and truncates for topological analysis were made using the corrected vector expressing HHAT-V5-His_6_ as a template.

For cysteine mapping topology analysis, selected cysteines were mutated to alanine by Q5 site-directed mutagenesis (New England Biolabs, Hitchin, UK). Q5 mutagenesis was also used for the introduction of the TEV protease site, ENLYFQG, in the HHAT-TEV mutants. For the production of the V5 topology clones, the V5-His_6_ epitope from the HHAT-V5-His_6_ construct was removed and replaced with a FLAG epitope (DYKDDDDK) by Q5 mutagenesis, followed by insertion of the V5 epitope (GKPIPNPLLGLDST) at the required sites.

For *N*-glycosylation analysis, an *N-*glycosylation site (DLT) was introduced by QuikChange II site-directed mutagenesis into the spacer region between the gene of interest cloning site, and the V5-His_6_ epitope of the empty destination vector pcDNA-DEST40 used for HHAT cloning Next, full-length HHAT, or the HHAT truncations HHAT-Δ157–493 and HHAT-Δ192–493 were amplified from the HHAT expression vector described above (15) and cloned into the modified destination vector using Gateway cloning, resulting in expression vectors carrying *N*-glycosylation, V5, and His_6_ epitopes at the C terminus. All clones were verified by sequencing.

##### SDS-PAGE and Immunoblotting

Separation of proteins was performed by SDS-PAGE using 10% or 15% Tris gels and Tris-glycine-SDS running buffer. Samples were prepared in NuPAGE® LDS 4× sample loading buffer (Invitrogen) and 10% (v/v) 2-mercaptoethanol (unless stated otherwise). The protein ladder used for comparison of molecular weight was Precision Plus Protein® Standards All Blue (Bio-Rad). Gels were run using the Mini-PROTEAN® Tetra cell system and power supply unit (Bio-Rad). Fluorescently tagged proteins were imaged (540 nm excitation and 595 nm emission, channel Cy3) using an Ettan DIGE imager (GE Healthcare), and images were analyzed with ImageQuant^TM^ TL software (GE Healthcare).

Proteins were transferred from SDS-PAGE gels to a PVDF membrane (Millipore) using a semidry transfer unit (Hoefer, Holliston, MA). Membranes were blocked with blocking solution (5% w/v milk powder (Marvel) dissolved in PBS) for 1 h at room temperature and washed with PBS-T (PBS containing 0.05% Tween 20 (Sigma)) and probed with appropriate antibodies. Antibodies and their sources were as follows: mouse anti-V5 monoclonal antibody (1:10,000 dilution, Invitrogen), mouse anti-His_6_ antibody (1:1000, AD1.1.10, R&D Systems, Abingdon, UK), rabbit anti-His_6_ antibody (1:1000, catalog no. ab137839, Abcam, Cambridge, UK), goat anti-Grp94 antibody (1:200 dilution, catalog no. C-19, Santa Cruz Biotechnology), and anti-calnexin-N terminus mouse monoclonal IgG1 (1:1000, catalog no. AF18, Sigma). Secondary antibodies used were as follows: goat anti-mouse IgG2a-HRP (1:20,000 dilution, Southern Biotech), goat anti-mouse IgG1-HRP (1:20,000, Southern Biotech), goat anti-rabbit-HRP IgG (1:20,000, Southern Biotech) and, after immunoprecipitations, mouse anti-rabbit IgG-HRP VeriBlot (1:1000, Abcam).

Visualization was carried out by enhanced chemiluminescence kit (Pierce ECL2 Western blotting substrate, Thermo Scientific) according to the instructions of the manufacturer and on an Ettan DIGE imager (GE Healthcare), excitation at 480 nm and emission at 530 nm (channel Cy2). Images were analyzed with ImageQuantTM TL software.

##### TEV Cleavage of ER Microsomes

HEK293a cells were transfected with wild-type or TEV mutant HHAT-V5-His_6_ constructs. After 48 h, cells were washed twice with cold HCN buffer (50 mm HEPES (pH 7.5), 150 mm NaCl, and 2 mm CaCl_2_) and pelleted at 800 × *g* for 2 min. Cells were passed through a 23-gauge needle ten times, and then intact cells and nuclei were pelleted at 800 × *g* for 5 min at 4 °C. The supernatant was then ultracentrifuged at 100,000 × *g* for 1 h to obtain microsomes, which were resuspended in ProTEV protease buffer (50 mm HEPES (pH 7.0), 0.5 mm EDTA, and 1 mm DTT), and protein concentration was determined using the DC protein assay (Bio-Rad). Because of lower expression levels of some mutants, an initial Western blot was performed on 5 μg of each microsome preparation to determine the amount of HHAT expression. The final concentration of microsome preparation treated with ProTEV was then normalized for HHAT expression. To every 20 μg of microsome suspension, 1 μl (5 units) of ProTEV Plus protease (Promega, Southampton, UK) was added, and then preparations were incubated at 25 °C for 4 h. The reaction was stopped with NuPAGE® LDS 1× sample loading buffer (Invitrogen). Proteins were then separated by SDS-PAGE and analyzed by immunoblotting with anti-V5 monoclonal antibody (1:10,000 dilution, Invitrogen). After probing for V5, blots were reprobed with anti-His_6_ (1:1000, catalog no. AD1.1.10, R&D Systems) antibody.

##### Metabolic Labeling with Alkynyl Palmitate (YnPalm) and Copper-catalyzed [3 + 2] Cycloaddition (CuAAC) Reaction

HEK293a cells were transfected with wild-type or cysteine-mutant HHAT-V5-His_6_ constructs. 36 h post-transfection, the medium was exchanged for feeding medium (DMEM, 3% FBS plus 50 μm alkynyl palmitate analog pentadec-14-ynoic acid (YnPalm) in DMSO (17), or the same volume of DMSO was used as vehicle control. After 16 h, cells were rinsed twice with ice-cold PBS and then lysed with 100 μl of lysis buffer (0.1% SDS, 1% Triton X-100, and EDTA-free Complete protease inhibitor (Roche Diagnostics) dissolved in PBS). Lysates were centrifuged at 16,000 × *g* for 10 min to remove insoluble material. The supernatant was collected and used for further experiments.

Cell lysates (20 μg of total proteins) were reacted with CuAAC reaction mixture containing azido-carboxytetramethylrhodamine (TAMRA)-PEG-Biotin (AzTB) (10 mm stock in DMSO) at 100 μm final concentration (17), CuSO_4_ (50 mm stock in water) at a final concentration of 1 mm, Tris(2-carboxyethyl)phosphine (TCEP) (50 mm stock in water) at a final concentration of 1 mm, and Tris[(1-benzyl-1*H*-1,2,3-triazol-4-yl)methyl]amine (TBTA) (10 mm stock in DMSO) at a final concentration of 100 μm. The reaction was vortexed for 1 h at room temperature, and EDTA (100 mm in water stock) was added to final concentration of 10 mm. Proteins were precipitated by addition of 4 volumes of methanol, 1 volume of chloroform, and 3 volumes of water. The sample was centrifuged for 5 min at 16,000 × *g*, and the pellet was washed twice with room temperature methanol. The protein precipitates were resuspended in 2% SDS in PBS and further diluted to 1 mg/ml of total protein concentration and a final SDS concentration of 0.2% with PBS. An aliquot of this sample was taken for SDS-PAGE analysis.

200 μg of proteins from cell lysate were immunoprecipitated with 1 μg of anti-V5 antibody. Following overnight incubation at 4 °C on a rotating wheel, 25 μl of Pureproteome protein G magnetic beads (Merck Millipore, Watford, UK) were added to each sample and incubated for 1 h at 4 °C on a rotating wheel. The beads were washed five times with lysis buffer and resuspended in 20 μl of PBS, and freshly premixed CuAAC reaction reagents at appropriate concentrations (as described above) were added. After 1 h of vortex mixing, the beads were washed five times with lysis buffer, mixed with NuPAGE® LDS 1× sample loading buffer (Invitrogen) and 10% (v/v) 2-mercaptoethanol, and incubated with vortexing at room temperature for 1 h. Proteins were separated by SDS-PAGE, and in-gel fluorescence was used to visualize YnPalm-modified proteins, which were then transferred to PVDF membranes and analyzed by immunoblotting with anti-His_6_ (1:1000, catalog no. ab137839, Abcam) antibody followed by mouse anti-rabbit IgG (HRP) VeriBlot (1:1000, Abcam) secondary antibody.

##### Hydroxylamine (NH_2_OH) Treatment of YnPalm-modified Proteins

After preparation of lysates from metabolic labeling of palmitoylated proteins with YnPalm (see section above), lysates were diluted 1:1 with 2 m NH_2_OH (pH 7.5) or 2 m Tris (pH 7.5). Samples were then incubated for 5 h at room temperature while mixing with a rotator wheel. HHAT proteins were then immunoprecipitated onto protein G magnetic beads with anti-V5 antibody and treated by CuAAC as described above. Proteins were separated by SDS-PAGE, and in-gel fluorescence was used to visualize YnPalm-modified proteins, which were then transferred to PVDF membranes (Millipore) and analyzed by immunoblotting with anti-His_6_ antibody.

##### mPEG Labeling of Cells

HEK293a cells were transfected with wild-type or cysteine mutant HHAT-V5-His_6_ constructs. After 48 h, cells were washed twice with ice-cold HCN buffer and pelleted at 800 × *g* for 2 min. Cells were then semipermeabilized in 0.02% digitonin or fully permeabilized in 1% Triton X-100 on ice for 20 min (in HCN buffer supplemented with a protease inhibitor mixture). In the experiments with microsomal fractions, 20 μg of microsomal fractions in cold HCN buffer with or without 0.2% Triton X-100 were used. 1 mm maleimide-polyethyleneglycol (mPEG) 5-kDa (Laysan Bio Inc.) was then added to each sample with or without 20 mm DTT for 30 min on ice, after which all samples were brought to a final concentration of 1% TX-100/20 mm DTT and incubated an for additional 15 min on ice. Lysates were clarified by spinning at 16,000 × *g* for 10 min, and the proteins in the supernatants were separated by SDS-PAGE. Samples were analyzed by immunoblotting with anti-V5, anti-His_6_, or anti-Grp94 antibodies.

##### HHAT V5 Topology Determination

Topology determination by V5 accessibility was performed as follows. 1.5 × 10^4^ HeLa cells were plated on 96-well imaging plates (ibidi) and transfected with wild-type or mutant HHAT-V5-FLAG constructs. After 48 h, cells were fixed with 3% paraformaldehyde in PBS (pH 7.5) for 10 min at room temperature, washed thoroughly, and then semipermeabilized in 0.02% digitonin/PBS or fully permeabilized in 0.2% Triton X-100/PBS on ice for 10 min. HHAT was visualized with mouse monoclonal anti-V5 IgG2A (1:300, Invitrogen) and rabbit polyclonal anti-DDDDK (1:300, catalog no. ab1162, Abcam), followed by Alexa Fluor 488-conjugated anti-mouse IgG2A and Alexa Fluor 555-conjugated anti-rabbit secondary antibodies (Invitrogen), respectively. As a control for the permeabilization of the cells, untransfected cells treated in the same way as the HHAT-transfected cells were stained for calnexin using two different antibodies, one against the N terminus of the protein, mouse monoclonal IgG1 (catalog no. AF18, 1:50, Santa Cruz Biotechnology), and one against the C terminus, rabbit polyclonal (catalog no. ADI-SPA-860, 1:100, Enzo Life Sciences), followed by Alexa Fluor 488-conjugated anti-mouse IgG1 and Alexa Fluor 555-conjugated anti-rabbit secondary antibodies, respectively. Imaging was performed using a Zeiss Axiovert 200 widefield microscope with a LD Achroplan ×40 0.60 KORR Ph2 objective and Hamamatsu OrcaER charge-coupled device camera in the FILM imaging facility. Colocalization analysis was performed using Volocity image analysis software (PerkinElmer Life Sciences). The two channels were thresholded by drawing a region of interest in a background area of the image. The mean intensity of the region for each channel was calculated, and this was set as the lower threshold. Regions of interest were then drawn around the ER regions of transfected cells, and then the Manders' colocalization coefficient was determined. Images from at least 40 cells from three independent experiments were used in the analysis.

##### In Vitro Expression of HHAT N-Glycosylation Mutants

*In vitro* protein expression of full-length HHAT, HHAT-Δ157–493, and HHAT-Δ192–493 truncations containing a C-terminal *N*-glycosylation motif was performed using the TnT quick-coupled transcription/translation system (Promega) on the basis of rabbit reticulocyte lysate with canine pancreatic microsomes. 40 μl of TnT master mix, 1 μg of plasmid template, 1 μl of methionine (1 mm), and 3.6 μl of canine pancreatic microsomes were mixed on ice. The volume was adjusted to 50 μl with RNase-free water, and reactions were incubated at 30 °C for 1.5 h.

##### Removal of Protein N-Glycosylation by Treatment with PNGase F

After *in vitro* protein transcription and translation, microsomal membranes were purified by diluting the components of the TnT reaction to 500 μl in PBS and carefully layered on top of 100 μl of 5% (w/v) sucrose in 1.5-ml tubes for ultracentrifugation (Beckman). The samples were centrifuged at 100,000 × *g* for 45 min at 4 °C to pellet the membranes. The supernatant was discarded, and the membrane pellet was resuspended in 10 μl of glycoprotein denaturing buffer supplied with the PNGase F kit (New England Biolabs) and heated at 100 °C for 10 min. The samples were incubated at 37 °C for 1 h with or without PNGase F (according to the instructions of the manufacturer). The reactions were stopped by addition of 4× NuPage LDS sample buffer (Invitrogen) and analyzed by immunoblotting against the V5 epitope.

##### Shh in Vitro Click Chemistry-based Palmitoylation Assay

Details of our Shh *in vitro* click chemistry-based palmitoylation assay will be reported fully elsewhere.^8^ Briefly, Shh residues 1–11 were synthesized by solid phase peptide synthesis attached to a C-terminal (PEG) biotin tag. YnPalm-CoA was synthesized from YnPalm and coenzyme A hydrate from yeast (Sigma) using standard 1,1′-carbonyl-diimidazole-coupling conditions. HEK293a cells were transfected with HHAT-V5-His_6_ (positive control), HHAT-H379A-V5-His_6_, HHAT-D339N-V5-His_6_, or mCherry (negative control) and grown to 70% confluency. Cells were lysed as described previously, and membrane fractions were collected via centrifugation at 100,000 × *g* for 1 h at 4 °C. Membrane fractions were solubilized in solubilization buffer (1% (w/v) *n*-dodecyl β-d-maltoside, 10 mm HEPES (pH 7.5), and 350 mm NaCl) for 1 h at 4 °C, and insoluble material was removed by centrifugation at 100,000 × *g* for 1 h at 4 °C. Solubilized HHAT-H389A and HHAT-D339N membrane fractions were normalized to HHAT-WT expression levels via α-His_6_ immunoblotting, and 3 μg of HHAT-WT or mCherry membrane fractions were used for *in vitro* palmitoylation. 1 μm YnPalm-CoA and 1 μm Shh(1–11)-biotin in reaction buffer (100 mm MES (pH 6.5), 20 mm NaCl, 1 mm DTT, and 0.1% (w/v) BSA) were incubated with solubilized membrane fractions for 30 min at room temperature in Reacti-Bind streptavidin-coated clear wells (Fisher). Wells were washed with 3× 200 μl of PBS-T + 1% BSA, followed by 3× 200 μl of reaction buffer. Wells were then subjected to CuAAC click chemistry functionalization with 10 μm azido-FLAG peptide, 1 mm CuSO4, 1 mm TCEP, 1 mm TBTA in PBS-T, and 0.1% BSA for 1 h at room temperature, washed with 3× 200 μl of PBS-T + 1% BSA, followed by 3× 200 μl of reaction buffer. Wells were probed with α-FLAG-HRP (Sigma) (1:20,000) in PBS-T and 0.1% BSA for 1 h at room temperature, washed with 3× 200 μl of PBS-T and 1% BSA, followed by 3× 200 μl of reaction buffer. Bound α-FLAG-HRP (Sigma) was visualized using BD OptEIA TMB reagent (BD Biosciences) according to the protocol of the manufacturer, and wells read for optical density at 450 nm. Samples were prepared in duplicate, and the experiment was repeated twice.

## RESULTS

### 

#### 

##### Bioinformatic Analysis of HHAT Topology

There are seven human HHAT isoforms (UniProt reference Q5VTY9). However, the canonical polypeptide sequence is isoform 1, which is 493 amino acids long. The topology for this isoform has been predicted previously using TMHMM 2.0 (18) to have eight TM helices (13). This model, however, had a number of inconsistencies in comparison with other MBOAT protein topologies, most significantly that the critical His-379 of the conserved MBOAT region of HHAT was predicted to be cytosolic, a feature that would be unique within this family of proteins and seems inconsistent with catalytic activity on Hh proteins in the ER lumen. A recent comparison of different topology prediction algorithms identified some as being superior for predicting structures of membrane proteins (19). The highest-scoring topology prediction algorithm was TOPCONS (20). Furthermore, the study that recently determined the topology of GOAT (21) utilized an advanced algorithm, MEMSAT-SVM (22), which employs a computer learning method and considers extra features, such as RLs and signal peptides.

**FIGURE 1.**
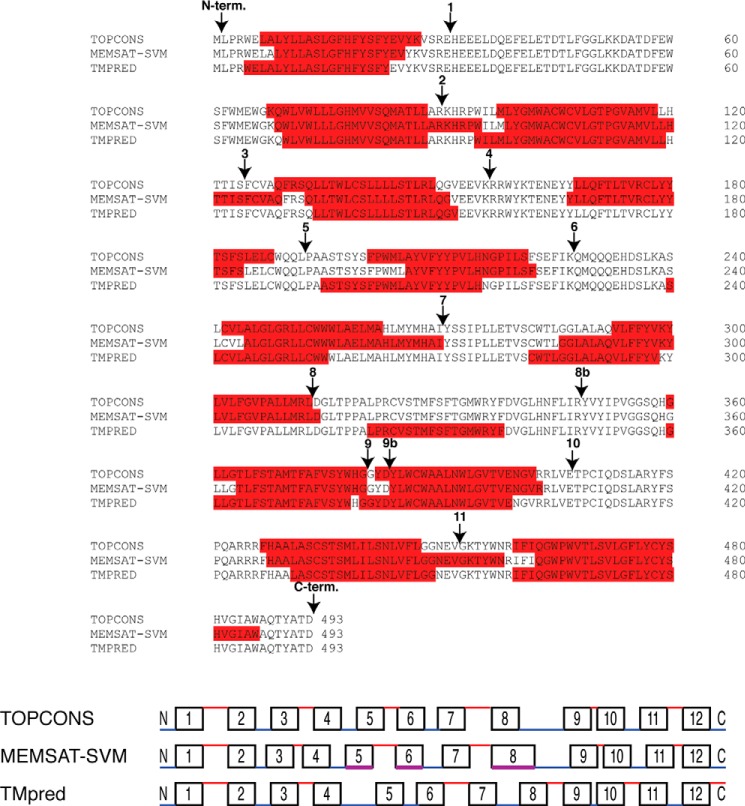
**Bioinformatic prediction analysis of HHAT sequences identifies a maximum of 13 predicted transmembrane domains.** Clustal Omega sequence alignment of human HHAT sequence with the TM predictions from three separate bioinformatic algorithms: TOPCONS, MEMSAT-SVM, and TMpred. Predicted TMs are highlighted in *red*, and loops are numbered in accordance with the epitope insertion analysis from our experiments. The TMpred algorithm predicted a helix within loop 8, and so mutants were made at two places in the loop between predicted TMs 8 and 9 to accommodate this prediction. These mutants were numbered 8 and 8b. In all models, the critical His-379 residue is predicted to be part of TM 9. Positions selected for epitope and TEV tag insertions used in this study are indicated by *arrows*. The *bottom panel* depicts the predicted topologies from each of the algorithms along with the predicted loop location. The *black boxes* depict the predicted TMs, whereas the *lines* depict the loop regions. Loops are colored *red* and *blue* depending on whether they were predicted to be luminal or cytosolic, respectively. In the MEMSAT-SVM diagram, TMs 5, 6, and 8 were predicted to be pore-lining helices, which are depicted in *magenta*. Loops and TMs are not drawn exactly to scale but do indicate relative size differences. *N-term.*, N-terminal; *C-term.*, C-terminal.

We performed topology predictions for HHAT sequences from 22 different species, identified on the NCBI RefSeq database (supplemental Fig. S1) (23) using both the TOPCONS and MEMSAT-SVM prediction algorithms (the data for human HHAT sequence are shown in Fig. 1; data for other species are not shown). Significantly, both prediction algorithms gave very similar results and were internally consistent across different species, with models predicted to contain either 11 or 12 TM domains in both algorithms.

Loop 8 was the longest, and another algorithm, TMpred (24), predicted an intervening TM, although with a low score. The other predicted TMs were similar to the TOPCONS and MEMSAT-SVM predictions (Fig. 1). TMpred predicts protein topology through several weight matrices on the basis of the statistical analysis from the SWISS-PROT TM protein database (release 25). To test this prediction in our experiments, we made two mutants within loop 8 (numbered 8 and 8b) for all experiments.

In all sequences analyzed, no N-terminal cleavable signal peptide was predicted either by MEMSAT-SVM or SignalP 4.0 (25). This was also confirmed experimentally (see below). Finally, in all topologies, the critical His-379 residue was predicted to be on the luminal side of the membrane at the border between predicted TM9 and loop 10.

##### HHAT Topology Determination by Tobacco Etch Virus (TEV) Protease Protection Assay on Purified ER Microsomes

On the basis of the predicted topology of human HHAT, a number of constructs were designed containing TEV protease recognition sites (ENLYFQG) within predicted loops, which also contained a V5-His_6_ epitope tag on the C terminus of the protein. The insertion sites are indicated in Fig. 1. TEV protease is a highly site-specific cysteine protease from tobacco etch virus. The optimum recognition sequence is ENLYFQG, and cleavage occurs between the Gln and Gly residues.

**FIGURE 2.**
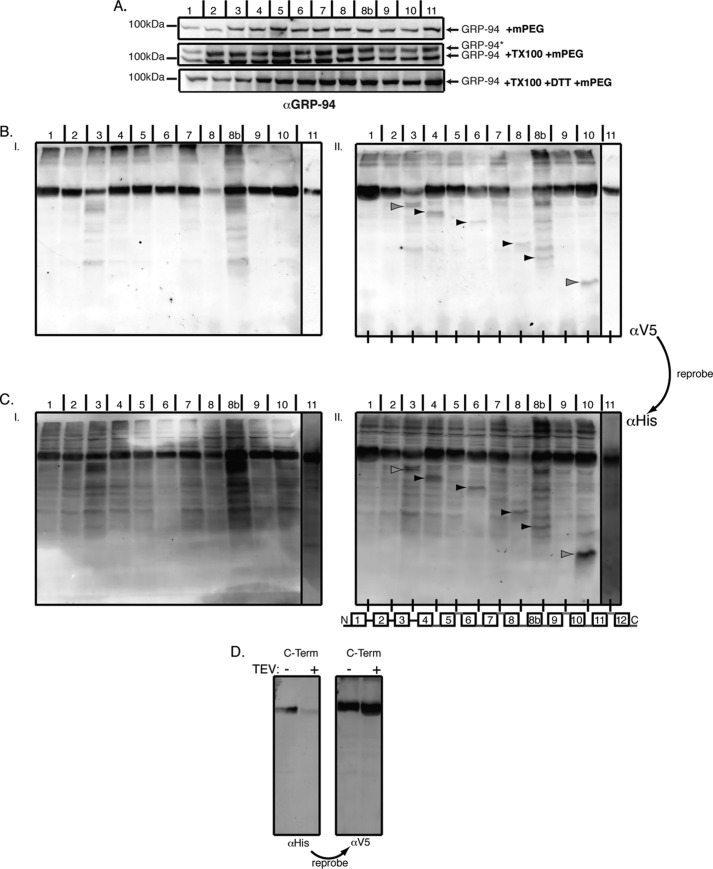
**Mapping of HHAT topology by TEV cleavage of microsomal preparations.** HHAT constructs containing a C-terminal V5-His_6_ epitope tag and an internal TEV protease tag at the indicated loops were transfected into HEK293a cells, and intact microsomal preparations were prepared by ultracentrifugation. *A*, to show that the microsomal preparations have intact ER membranes, mPEG, a hydrophilic reagent of 5 kDa that reacts with -SH groups, did not react with the non-disulfide-bonded cysteine in the ER luminal protein GRP94 when it was added to 10 μg of the microsomal preparations (*top panel*). After treatment of the microsomes with 0.2% Triton X-100 (*TX100*, *center panel*), GRP94 is modified by mPEG, and the reaction could be blocked by the reducing agent DTT (*bottom panel*). GRP94* indicates the position of the modified proteins. *B*, microsome preparations were treated with TEV buffer (*I*) or Pro-TEV protease (see “Experimental Procedures”) (*II*) for 4 h at 25 °C with agitation. The reaction was then stopped with sample buffer, and samples were analyzed by SDS-PAGE and probed first with anti-V5 antibody and then reprobed with anti-His_6_ antibody. Because the microsomal preparations are intact, any TEV cleavage sites on luminal loops will be inaccessible to the Pro-TEV protease. However, any cytosolic sites will be accessible to the protease, and so the protein will be cleaved and produce a shorter C-terminal fragment that will be identified by antibody labeling. *C*, using this method, the HHAT topology model (*II*, *bottom panel*) was produced. *Red lines* indicate that the TEV site was inaccessible and, hence, luminal. *Blue lines* indicate that the TEV site was accessible and, hence, cytoplasmic. Cleaved bands are indicated by *black arrowheads*, whereas *gray arrowheads* indicate potential cleaved products. It was unclear whether mutants 1/2 and 2/3 produced any cleavage product. Immunoblots shown are representative results from five independent experiments. *D*, a construct containing the TEV protease tag in the linker sequence between the C-terminal (*C-term.*) V5 epitope and the His_6_ epitope tag was used to examine the topology of the C terminus of HHAT. Specifically, if the C terminus is cytosolic and accessible to TEV protease cleavage, then reactivity of the protein with a His_6_ antibody would be lost after treatment with Pro-TEV, whereas reactivity with the V5 antibody should be retained. This is what was observed in the experiments with this construct, indicating that the C terminus is cytosolic.

We isolated intact microsomes containing ER membranes from cells expressing HHAT-TEV constructs and treated these preparations with ProTEV protease *in vitro* (Fig. 2). We first confirmed that the ER membrane was intact by checking the ability of the thiol-reactive reagent mPEG to modify the non-disulfide-bonded cysteine in the ER luminal protein GRP94 (Fig. 2*A*) (26). Isolated microsomes treated with mPEG had only a single GRP94 band at ∼100 kDa (Fig. 2*A*, *top blot*). However, when 0.2% Triton X-100 was added during mPEG treatment to permeabilize the membrane, a second mPEG-modified band appeared (Fig. 2*A*, *center blot*), and this band disappeared when treated at the same time with 20 mm DTT, which competes with mPEG reacting with the cysteine in GRP94 (Fig. 2*A*, *bottom blot*). Having confirmed that the microsomes were intact, we proceeded with protease treatment.

Protein levels were adjusted to achieve equal levels of mutant HHAT expression, and the amount of ProTEV protease was adjusted for protein concentration (see “Experimental Procedures”). In the absence of ProTEV protease (Fig. 2, *BI* and *CI*), all HHAT constructs migrated as single ∼50-kDa bands. However, with the addition of ProTEV, additional bands appeared, indicating successful cleavage of some of the HHAT-TEV constructs. This is particularly apparent from the sizes of these additional bands, which correspond to the expected size of tagged C-terminal peptides produced on cleavage of the proteins. Therefore, proteins with TEV sites in loops close to the N terminus of the protein produce larger cleavage products than proteins with TEV sites close to the C terminus of the protein. Using this approach, we were able to verify that loops 4, 6, 8, and 10 were cytosolic (Fig. 2, *BII* and *CII*, cleavage products are indicated by *black arrowheads*), whereas the C terminus of the protein was also cytosolic because a HHAT-TEV construct in which the TEV recognition sequence was placed between the C-terminal V5 and His_6_ epitopes showed a loss of αHis_6_ immunoreactivity (Fig. 2*D*). Mutants HHAT-3 TEV and HHAT-8b TEV showed the appearance of a band in the presence of TEV, but because of background bands obscuring the clear appearance of the cleaved product we were unable to determine the loop topology reliably (Fig. 2, *BII* and *CII*, potential cleavage products are indicated by *gray arrowheads*). Loops 5, 7, 9, and 11 showed no evidence of TEV cleavage and, therefore, appear to be luminal. With regard to the first two loops of the protein, because of the size of the cleavage products coinciding with the size of ProTEV and the relatively lower immunoreactivity of the cleavage products using the anti-V5 antibody (compare Fig. 2, *BII* and *CII*), it was not clear whether the protein was cleaved. All mutants cleaved when cells were fully permeabilized with dodecyl maltoside, showing that the cleavage sites were functional (data not shown). These experiments are consistent with a ten-TM model for HHAT topology with two RLs (RL3 and RL6) and a cytosolic C terminus (Fig. 2, *CII*, diagram below the blot).

##### HHAT Mapping by Epitope Immunoreactivity in Selectively Permeabilized Cells

**FIGURE 3.**
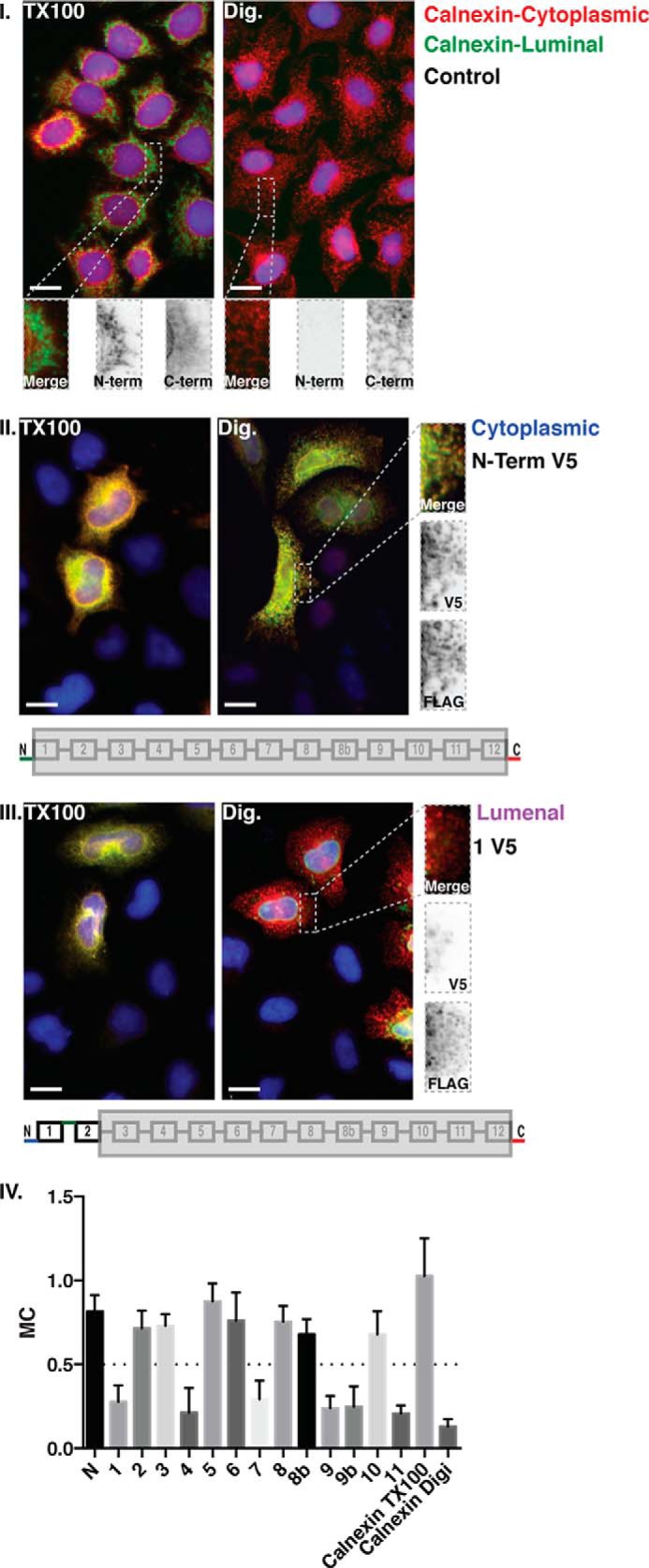
**Mapping of HHAT topology by V5 epitope indirect immunofluorescence in selectively permeabilized cells.** HHAT constructs containing a C-terminal FLAG epitope tag and an internal V5 epitope tag at the indicated loops were transfected into HeLa cells plated on 96-well imaging plates. Cell membranes were either permeabilized with 0.2% Triton X-100 (*TX100*) or with 0.04% digitonin (*Dig*). Permeabilization with digitonin maintains the ER membrane intact, whereas permeabilization with Triton X-100 fully permeabilizes all membranes of the cell. FLAG and V5 epitopes were stained with specific primary antibodies, followed by secondary antibodies with 555-nm (*red*) or 488-nm (*green*) excitation wavelengths, respectively. To control for the selective permeabilization of the ER membrane, untransfected cells were selectively permeabilized and stained for Calnexin using two different antibodies, one that binds to an epitope on the luminal N terminus of the protein and another that bind to an epitope on the cytosolic C terminus of the protein (*I*). Only two conditions are shown, for the V5 epitope in the N terminus of the protein (*II*) and in predicted loop 1 (*III*). The N and C termini of HHAT were both cytosolic (*II*), therefore the FLAG epitope will always be stained regardless of the detergent used for permeabilization. Images were then analyzed using Volocity image analysis software to determine the colocalization coefficient of the two channels. The *insets* show a higher magnification of the indicated regions. The histogram (*IV*) shows mean Manders' coefficients (*MC*) for colocalization of the red (555-nm) channel with the green (488-nm) channel for different HHAT mutants and for the Calnexin control (*n* > 40 cells, data are mean ± S.D.). High Manders' coefficients indicate better colocalization of FLAG-tagged proteins with V5-tagged proteins. Values less than 0.5 indicate low colocalization. Identical exposures and image normalization for both permeabilizations ensure a fair side-by-side comparison. The bottom panels of *II* and *III* illustrate the positions of the stained epitopes. Images are examples of each condition from at least three independent experiments. *Scale bars* = 15 μm.

We wanted to complement the results obtained with TEV protease mapping with an alternative method, ideally in a cellular context and in a manner that would give a definite result for loops near the termini of HHAT and for the N and C termini themselves. We decided to pursue an epitope mapping strategy in selectively permeabilized cells using indirect immunofluorescence. We generated HHAT constructs that were C-terminally tagged with a FLAG tag (DYDDDDK) and had a V5 epitope (GKPIPNPLLGLDST) inserted at the same points at which the TEV protease site had been inserted, as well as an N-terminal V5-tagged version (see Fig. 1). HeLa cells expressing these constructs were fixed and treated with 0.2% Triton X-100 to fully permeabilize the cell membranes or treated with 0.04% digitonin, which selectively permeabilizes the plasma membrane of the cells but leaves the ER membrane intact (21, 26). Cells were then stained for both FLAG and V5 epitopes by indirect immunofluorescence (Fig. 3). As a control for the permeabilization protocol, cells were stained with two different Calnexin antibodies, one that recognizes the N terminus of the protein and another that recognizes the C terminus of the protein. Calnexin is a type I transmembrane domain protein that is abundant in the ER and functions as a chaperone for secretory glycoproteins (27), the N terminus of which is luminal and the C terminus cytoplasmic (28). Therefore, in digitonin-treated cells, the N terminus-specific antibody should not react with the protein, but the C terminus specific antibody should react regardless of treatment (Fig. *3**I*). A similar analysis was performed using the HHAT-V5-FLAG constructs in permeabilized and semipermeabilized cells (the data for the two mutants are shown in Fig. 3, *II* and -*III*). Images were collated from at least three separate experiments, and Manders' correlation coefficient was calculated for each mutant in the digitonin-treated condition to determine whether the two probes were on the same side of the ER membrane (Fig. 3*IV*).

As expected from the TEV experiments, the C terminus of HHAT was cytosolic for all HHAT constructs. This also confirmed that none of the insertions caused the TMs to flip (Fig. 3 and data not shown). The N terminus was also cytosolic, as predicted by both TOPCONS and MEMSAT-SVM (Fig. 3*II*). The staining also confirmed that the protein does not contain an N-terminal cleavable signal peptide because the V5 tag would have been removed in that case. The results for the other mutants correspond mostly, but not completely, to the TEV protease experiments. Specifically, loops 2, 3, 5, 6, 8, and 10 all appeared to be cytosolic, whereas loops 1, 4, 7, 9, and 11 were luminal (Fig. 5, *top panel*). In the case of the loop 9 mutant, we decided to make a second mutant in which the V5 epitope was shifted C-terminally by three amino acids (HHAT-9b V5-FLAG) to correct for any potential masking of the epitope from being in close proximity to a predicted TM domain. However, the results still suggested that the loop was luminal. The results of the epitope mapping showed a very good overlap with the TEV experimental model. Noteworthy exceptions were loops 4 and 5, which the TEV experiments placed as cytosolic and luminal, respectively, whereas, in the epitope mapping, the opposite orientation was seen.

**FIGURE 4.**
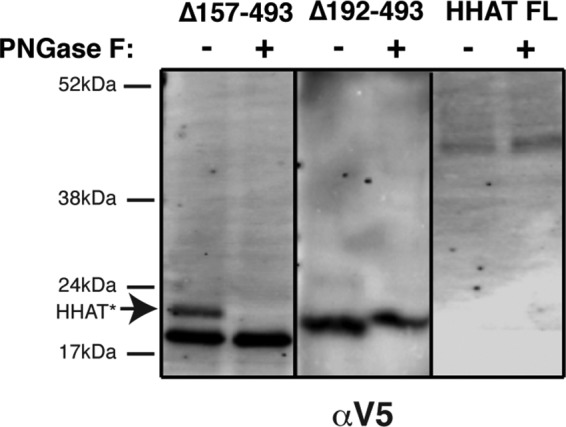
***N*-glycosylation analysis of full-length HHAT, HHAT-Δ157–493, and HHAT-Δ192–493 truncation mutants containing a C-terminal *N*-glycosylation motif.** *In vitro* translation/translocation of HHAT truncations ending in predicted loop regions 4 (Δ157–493 mutant) and 5 (Δ192–493 mutant) or full-length (*FL*) HHAT cDNA C-terminally tagged with an *N*-glycosylation site and a V5 epitope. Luminal *N*-glycosylation sites become glycosylated, and the covalent attachment to the protein can be detected by a shift in molecular weight on an SDS-PAGE gel. Following expression, lysates were treated with PNGase F, an endoglycosidase that cleaves *N*-glycans between the innermost sugar moiety and Asn, removing the shift in molecular weight of glycosylated proteins, or left untreated. Lysates were analyzed by SDS-PAGE on 7.5% gels, followed by immunoblotting with anti-V5 antibody. HHAT*, glycosylated HHAT protein.

**FIGURE 5.**
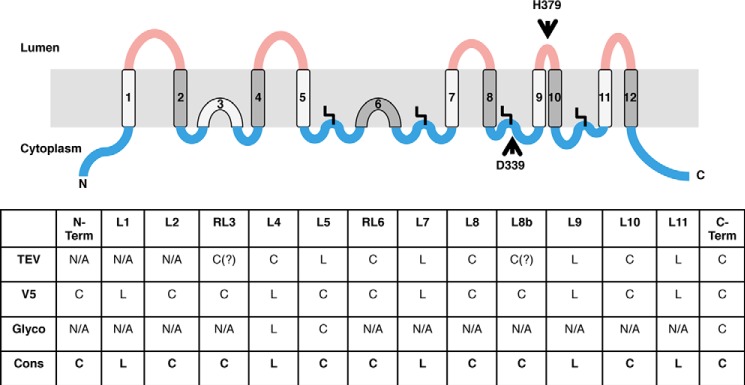
**HHAT topology experimental consensus model indicating ten TMs and two RLs.** Shown is the consensus HHAT topology model, including palmitoylation modifications, on the basis of the data shown in the *bottom panel* which summarizes the results from the three different experiments, indicating whether a loop is luminal (*L*) or cytoplasmic (*C*). The consensus (*Cons.*) model is indicated in the *bottom row*. Our data indicate that predicted TM 3 and TM 6 are likely to be RLs. The cytosolic loops containing cysteines that are palmitoylated display the fatty acid modification as a *bent line*. Relative loop length is approximated by the size of the loops but not precisely scaled. Cytosolic loops are colored *blue*, and luminal loops are *red*. The positions of the two conserved MBOAT residues, Asp-339 and His-379, are indicated by *arrows*.

To resolve this discrepancy, C-terminal truncation mutants of HHAT containing the first four or five predicted TMs of HHAT and a C-terminal N-glycosylation motif followed by the V5-His_6_ epitope were constructed and expressed by *in vitro* translation in the presence of microsomes. Lysates were then analyzed by Western blotting to see whether the C terminus of the protein was glycosylated or not (Fig. 4). This method supported a luminal location for loop 4 and a cytosolic location for loop 5, confirming the V5 epitope mapping result (Fig. 3*IV*). We also confirmed, in the same way, that the C terminus of full-length HHAT is cytosolic (Fig. 4). On the basis of the TEV protease mapping, the epitope mapping on selectively permeabilized cells, and the glycosylation of HHAT truncations, a consensus topology model can be drawn up in which HHAT has ten TM domains and two RLs (RL3 and RL6, Fig. 5), whereas His-379 is close to the luminal side of the ER, and Asp-339 is cytosolic.

##### Cytoplasmic Cysteine Accessibility Suggests Cysteine Modification and a Stabilizing Role for Cys-324

HHAT has 14 cysteines, and, on the basis of the consensus experimental topological model of HHAT described above (Fig. 5), HHAT is predicted to have three luminal cysteines, Cys-126, Cys-139, and Cys-282, and four cytosolic cysteines, Cys-188, Cys-242, Cys-324, and Cys-410, whereas the rest of the cysteines are embedded in the hydrophobic core of the membrane.

**FIGURE 6.**
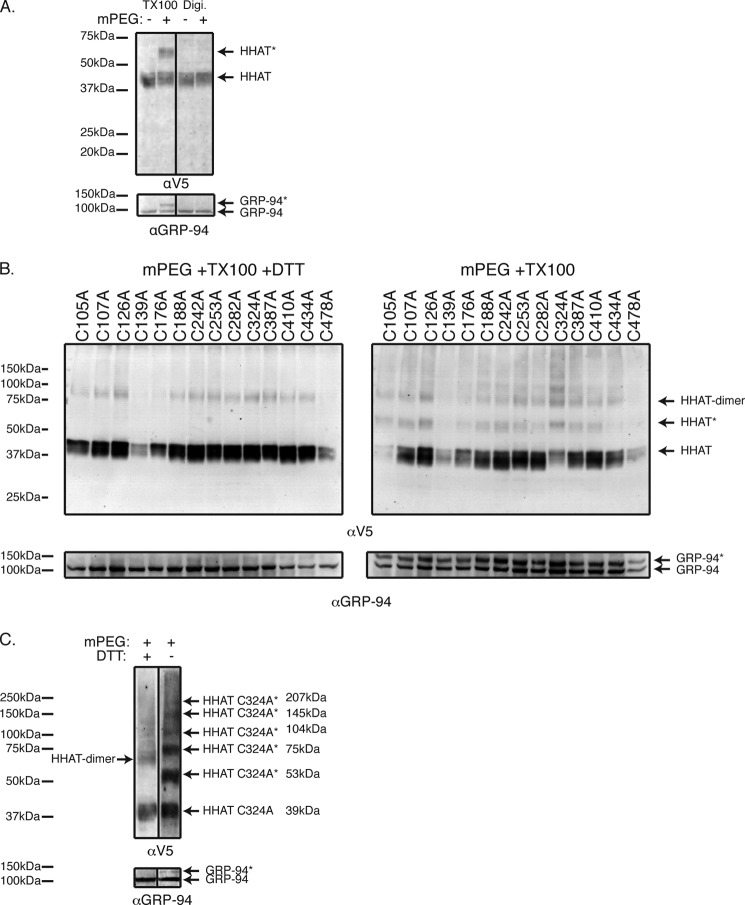
**HHAT cysteine mapping in selectively permeabilized cells.** *A*, HEK293a cells expressing HHAT-V5-His_6_ were permeabilized with 0.04% digitonin (*Digi.*) or 1% Triton X-100 (*TX100*) as indicated. Modification was carried out for 30 min at 4 °C with 1 mm mPEG. Samples were analyzed by SDS-PAGE on 7.5% gels, followed by immunoblotting with antibodies to V5 (*top panel*) or GRP94 (*bottom panel*). *B*, HEK293a cells expressing the indicated HHAT single cysteine mutants were permeabilized with 1% Triton X-100. Modification was carried out for 30 min at 4 °C with 1 mm mPEG. Where indicated, DTT was present during the reaction. Samples were analyzed by SDS-PAGE on 7.5% gels, followed by immunoblotting with antibodies to V5 (*top panels*) or GRP94 (*bottom panels*). The immunoblots shown are representative results from four independent experiments. *C*, HEK293a cells expressing HHAT C324A mutant were permeabilized with 1% Triton X-100. Modification was carried out for 30 min at 4 °C with 1 mm mPEG. Where indicated, DTT was present during the reaction. Samples were analyzed by SDS-PAGE on 7.5% gels, followed by immunoblotting with antibodies to V5 (*top panel*) or GRP94 (*bottom panel*). HHAT* and GRP94* indicate the positions of the modified proteins.

To examine this prediction, we transfected cells with the wild-type HHAT-V5-His_6_ construct and either fully permeabilized the cells with 1% Triton X-100 or semipermeabilized the cells with 0.04% digitonin and treated the cells with mPEG (Fig. 6*A*). The ER membrane was intact in digitonin-treated cells because GRP94 was not modified. Surprisingly, in fully permeabilized cells, there was only a SDS-PAGE shift of HHAT-V5-His_6_ corresponding to ∼15 kDa (analysis of four experiments indicates a shift of 14.1 ± 0.84 kDa), indicating that only three cysteines were modified by mPEG (which has a molecular mass of ∼5 kDa). Even more surprisingly, in the semipermeabilized cells, no band shift was seen, indicating that all three tagged cysteines were luminal. This supports our hypothesis that three cysteines of HHAT would be in the lumen of the ER but raised the question why no cytosolic cysteines were accessible.

It is possible that cytosolic cysteines are not accessible to mPEG through steric hindrance from the secondary, tertiary, or quaternary structure of the cytosolic loops. Another possibility is that the -SH groups of the cysteines are not available to react with mPEG because of another modification or formation of a disulfide bond with neighboring cysteines, although this is unlikely for cytosolic cysteines because of the reducing environment of the cytoplasm. This was also excluded by running HHAT on non-reducing gels in which we did not see altered mobility, which would be evidence of disulfide bond formation (data not shown). Therefore, either the cysteines were inaccessible or they were modified in some other way.

We performed an alanine scan of all cysteines followed by mPEG labeling in fully permeabilized cells to see whether we could identify the cysteines that were being labeled with mPEG (Fig. 6*B*). None of the single cysteine mutations resulted in a quantifiable reduction of the mPEG modification of HHAT. Unusually, the cysteine mutant C324A, when tagged with mPEG, gave rise to multiple bands of increasing size (Fig. 6, *B*, *lane 10*, *right panel*, and *C*), suggesting that, by mutating this single cysteine, other cysteines within the protein somehow become accessible and/or multimer formation is promoted.

##### HHAT Is Palmitoylated on Multiple Cytoplasmic Cysteines

The inaccessibility of HHAT cytosolic cysteines to the mPEG alkylating reagent and the lack of disulfide bonds in the protein suggested that the cysteines are perhaps modified in some other way. We hypothesized that HHAT is palmitoylated, partly on the basis of studies showing that the DHHC PATs form an autoacylated intermediate (29) before acylating their substrate proteins. Furthermore, cytosolic juxtamembrane cysteines of transmembrane proteins are frequently palmitoylated. Therefore, we decided to examine whether HHAT was acylated.

**FIGURE 7.**
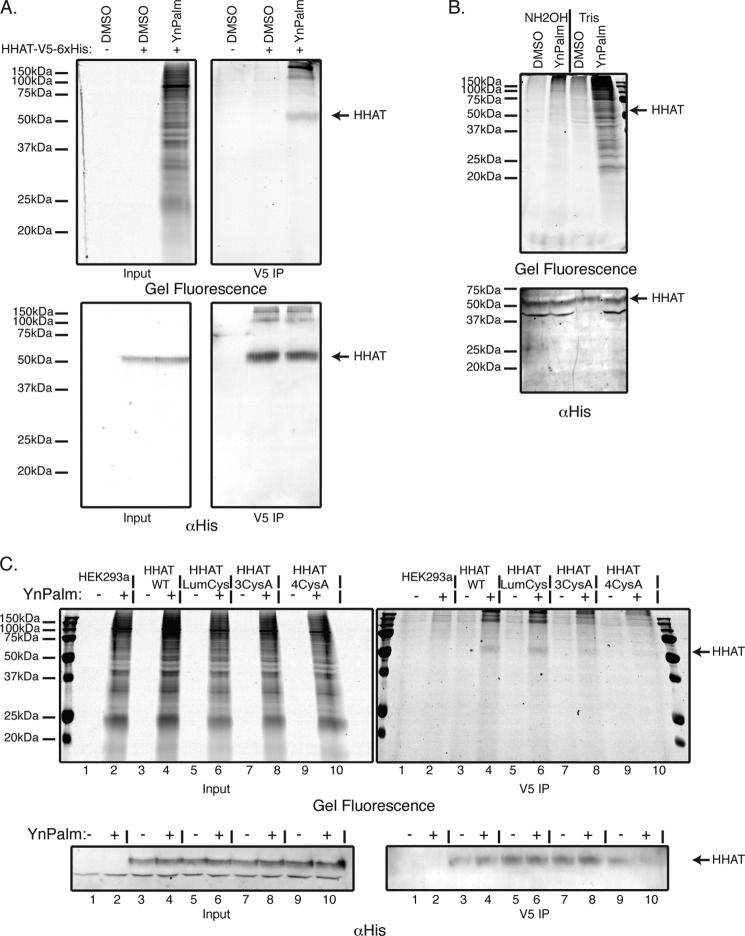
**HHAT is palmitoylated on multiple cysteines via thioester linkage.** *A*, HEK293a cells were transfected with a construct expressing the WT human HHAT cDNA C-terminally tagged with V5 and His_6_ epitopes. Cells were then fed overnight with 50 μm palmitic acid analog with a clickable alkyne moiety (YnPalm) or with vehicle (0.1% DMSO). Cells were lysed, and HHAT was immunoprecipitated (*IP*) using anti-V5 antibody. Immunoprecipitated proteins or lysates were ligated by CuAAC to AzTB. Tagged proteins were separated on 15% SDS-PAGE gels and analyzed by in-gel fluorescence (*top panels*) and immunoblotting with antibody against His_6_ (*bottom panels*). *B*, lysates were prepared as in the previous experiment. However, before immunoprecipitation, lysates were either treated with 1 m NH_2_OH (pH 7.5) or 1 m Tris (pH 7.5) for 5 h at room temperature, as described under “Experimental Procedures.” Lysates were then precipitated by chloroform/methanol precipitation, resolubilized in 0.2% Triton X-100 in PBS, immunoprecipitated with αV5 antibody, ligated by CuAAC to AzTB, and analyzed by SDS-PAGE in-gel fluorescence (*top panel*) and anti-His_6_ immunoblotting (*bottom panel*). *C*, HEK293a cells were transfected with constructs expressing WT human HHAT cDNA, the luminal cysteine mutant HHAT-LumCys, or the cytosolic cysteine mutants HHAT-3CysA or HHAT-4CysA. Cells were then fed overnight with 50 μm YnPalm or 0.1% DMSO. Cells were then lysed, immunoprecipitated (*IP*) with anti-V5 antibody, ligated by CuAAC to AzTB, and analyzed by SDS-PAGE in-gel fluorescence (*top panels*) and anti-His_6_ immunoblotting (*bottom panels*).

HEK293a cells overexpressing human HHAT-V5-His_6_ were metabolically labeled with YnPalm (17), cells were lysed, and HHAT was immunoprecipitated via its C-terminal V5 tag onto protein G beads. Precipitated proteins were then subjected to CuAAC ligation to the azide-containing trifunctional capture reagent AzTB (17). Proteins were separated by SDS-PAGE, and palmitoylated proteins were then identified by in-gel fluorescence (Fig. 7*A*, *top panels*) and verified by anti-His_6_ immunoblotting (Fig. 7*A*, *bottom panels*). As seen in the input samples (Fig. 7*A*, *top left panel*), there are multiple bands corresponding to all the proteins in the palmitoylome of the cell. However, after V5 immunoprecipitation (Fig. 7*A*, *top right panel*), a major band is visible at 50 kDa, corresponding to palmitoylated HHAT. Furthermore, treatment of the lysates with hydroxylamine (NH_2_OH) at neutral pH, which cleaves thioester bonds, before V5 pulldown and CuAAC ligation, led to a loss of the HHAT palmitoylation signal (Fig. 7*B*), confirming that HHAT is palmitoylated via a thioester bond on at least one cysteine residue.

We used the cysteine-to-alanine mutants discussed above in similar metabolic labeling experiments and in Acyl-RAC pulldown experiments (30). However, no single cysteine resulted in significant decreased pulldown, suggesting that multiple cysteines were being acylated (data not shown).

On the basis of our experimentally determined topology of HHAT (Fig. 5), four cysteines, Cys-188, Cys-242, Cys-324, and Cys-410, are expected to be on the cytosolic side of the ER membrane, where palmitoylation by DHHC PATs is expected to occur. We made two multiple cysteine-to-alanine mutants in which either all four cysteines were mutated to alanine (4CysA), or just three of the cysteines were mutated (3CysA), leaving out Cys-188, which is predicted to be within a reentrant loop and therefore may not be accessible to mPEG modification (see section above). These mutants were metabolically labeled with YnPalm and analyzed by anti-V5 precipitation and CuAAC ligation to AzTB (Fig. 7*C*). In the HHAT-3CysA mutant, there was a decrease in the palmitoylated HHAT signal, although a faint band was still visible in the in-gel fluorescence (Fig. 7*C*, *right panel*, compare *lane 4* with *lane 8*). However, mutation of all four cysteines to alanine in the HHAT-4CysA mutant, led to a complete loss of YnPalm signal (compare *lane 4* with *lane 10*), indicating that all four cysteines on the cytosolic side of the protein are palmitoylated. Significantly, a mutant protein in which all the predicted luminal cysteines were mutated to alanine, HHAT-LumCys mutant, was still palmitoylated to an equal extent as the wild-type protein (Fig. 7*C*, compare *lane 4* with *lane 6*).

**FIGURE 8.**
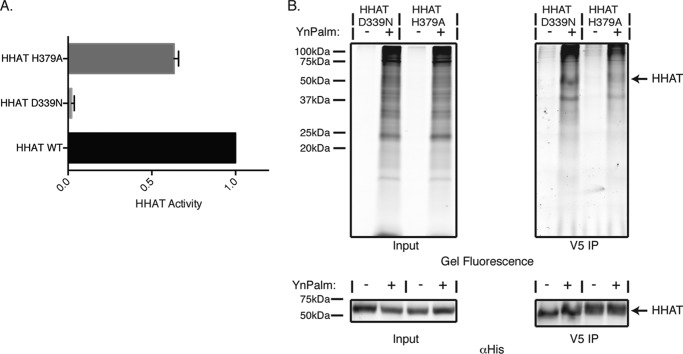
**His-379 is implicated in HHAT palmitoylation.** *A*, HHAT activity for the D339N and H379A mutants was determined by measuring the ability of the purified and solubilized HHAT mutant proteins to palmitoylate a Shh peptide using an in-house developed *in vitro* click chemistry-based palmitoylation assay. HHAT-D339N-V5-His_6_ showed only ∼3% activity compared with the WT, whereas the HHAT-H379A-V5-His_6_ mutant had ∼63% activity compared with the WT. Data are presented as mean ± S.D. and normalized to HHAT-WT-V5-His_6_. *B*, HEK293a cells were transiently transfected with HHAT-D339N-V5-His_6_ or HHAT-H379A-V5-His_6_ mutant constructs. Cells were then fed overnight with 50 μm YnPalm or with 0.1% DMSO. Cells were lysed and HHAT was immunoprecipitated (*IP*) using a V5 antibody. Immunoprecipitated proteins or lysates were ligated by CuAAC to AzTB. Tagged proteins were separated on 15% SDS-PAGE gels and analyzed by in-gel fluorescence (*top panel*) and immunoblotting with antibody against the His tag (*bottom panel*). Gels and immunoblots shown are representative results from two independent experiments.

Finally, we examined whether HHAT activity was important for its palmitoylation. The HHAT mutants D339N and H379A have significantly reduced activity (13), with D339N showing a severe loss of activity, whereas H379A retained ∼60% activity of WT HHAT (Fig. 8*A*). HEK293a cells overexpressing HHAT-D339N or HHAT-H379A were metabolically labeled with YnPalm, and HHAT palmitoylation was examined by in-gel fluorescence (Fig. 8*B*). These experiments showed that HHAT-D339N was still palmitoylated. However, HHAT-H379A was only weakly palmitoylated, indicating that the conserved His-379 residue of the MBOAT region of HHAT is important for the palmitoylation of HHAT and that this may be critical for HHAT function.

## DISCUSSION

In this study, we determined the topology of HHAT by TEV protease and V5 epitope mapping in microsome preparations and selectively permeabilized cells, respectively, and we also showed that HHAT is multipalmitoylated, which may affect protein function.

The model we developed (Fig. 5) consists of ten TMs and two RLs with cytosolic N- and C termini, the conserved His-379 residue from the MBOAT domain at the luminal tip of TM-9, and the critical Asp-339 in a large cytosolic loop that contains a palmitoylated Cys. This model is significantly different from the previously proposed model that consisted of eight TMs and in which the His and Asp residues were in a single large cytosolic loop (13). Our experimentally validated model shows a good correlation with the predicted topologies of both TOPCONS and MEMSAT-SVM algorithms, the biggest discrepancy being that neither model predicted any RLs. This is perhaps not surprising because, despite both TOPCONS and MEMSAT-SVM containing features to identify RLs, the accuracy of predicting such regions is significantly lower (22, 31). Furthermore, we do not know to which class of RL they belong (31) or whether the RLs actually enter the membrane at all or rather form some globular domain.

To date, the topology of two other members of the MBOAT family of proteins that acylate proteins, Gup1p and GOAT, have been examined in any great detail. Both had complex models that were, however, similar to the HHAT topology in our study. Gup1p, a yeast protein that acylates (with C26:0) glycosylphosphatidylinositol (GPI)-anchored proteins, was found to have nine TMs and three RLs (32), whereas GOAT has been shown recently to contain 11 TMs and one RL (21). In both cases, the invariable His residue was luminal, and the conserved Asn was cytosolic, similar to what we observe for HHAT, further enhancing the theory that all MBOATs have this structure for their proposed active sites (21, 32, 33). HHAT is unusual compared with the other MBOAT proteins in that mutation of the invariable His residue of the MBOAT region does not completely abolish the catalytic activity of the protein (Fig. 8*A*) (13). This suggests that HHAT may function in a different manner than other members of the family. However, our model does not indicate, at least from a topological point of view, that the active site is different from that of Gup1p or GOAT. Alternatively, substrate specificity may underlie this difference. GOAT and Gup1 acylate serine and glycerol, respectively, both oxygen nucleophiles, whereas HHAT acylates a thiol. In GOAT and Gup1, His may act as a base to enhance the nucleophilic character of the hydroxyl group via activation/deprotonation, but, in the case of HHAT, the Shh cysteine may be a sufficiently good nucleophile to attack palm-CoA without activation. However, similar to what has been shown for other MBOAT proteins, mutation of the conserved Asp in HHAT does abrogate function completely (Fig. 8*A*) (13). The cytosolic location of this residue suggests that it is not directly involved with the palmitoylation of Hh proteins and likely is involved with either substrate recognition or HHAT stability. Although Asp-339 is cytosolic, it is in the same loop as the palmitoylated Cys-324, which would insert into the bilayer and have the effect of bringing this loop back up to the membrane surface where Asp-339 could interact with substrates.

RLs can have multiple functions within proteins, especially in pore-forming transport channels. In aquaporins, two RLs are surrounded by six TMs, therefore lining the channel and providing selectivity to the transporter specifically for water molecules (34), whereas, in the glutamate transport proteins, a RL determines substrate binding within the pore (35). RLs can also stabilize the subunits of different channels through electrostatic interactions (36). It will be important to examine whether RL6 in both GOAT and HHAT functions in substrate recognition, especially because the acyl-CoA is negatively charged, and it may be possible that RLs can create regions of positive electrostatic potential to aid the interaction (36). These regions may also affect the formation of HHAT multimers. Although GOAT is suggested to exist and function as a monomer (21), HHAT does form non-covalent dimers in cells (Fig. 6*B*) and when the protein is purified *in vitro* (data not shown). It is unclear whether the protein functions as a monomer or as a dimer. However, recent studies in the DHHC PATs revealed that DHHC2 and DHHC3 form inactive dimers and that dimerization is inhibited by the autoacylation (37).

Using metabolic labeling experiments and multiple Cys-to-Ala mutants of HHAT, we further show that HHAT is *S*-palmitoylated on four cytosolic Cys residues. Surprisingly, the impaired activity mutant HHAT H379A is only weakly palmitoylated, indicating that palmitoylation of HHAT is important for its function. This could imply that the role of His-379 in HHAT would be that of a structural regulator in the protein rather than being involved in catalytic activity. Unlike the DHHC PATs, where the importance of the DHHC motif and, particularly, of the Cys residue of the motif to the acylation of their target proteins is better understood, very little is known about how the invariant His residue of the MBOAT proteins functions in catalysis of the acylation of lipids and proteins. In the DHHC PATs, autoacylation at the Cys residue of the motif occurs upon interaction with the acyl-CoA to form a transient acyl-enzyme transfer intermediate before transferring the acyl group to the protein substrate (29). It is likely that the palmitoylation of HHAT is not directly involved in the *N*-palmitoylation of Hh proteins in a comparable manner with DHHC proteins because HHAT acylation sites and Hh acylation occur on opposite sides of the membrane. However, the palmitoylation of HHAT may modulate protein function and/or structure in other ways that may ultimately affect Hh protein palmitoylation.

A recent study showed that the closely related MBOAT protein PORCN, which attaches a palmitoleoyl moiety to the Wnt family of proteins, is also palmitoylated (38). This study suggests that PORCN was palmitoylated on a single Cys conserved only in primates, specifically Cys-187 of human PORCN, and that palmitoylation of PORCN resulted in a reduction of palmitoleoylation and activity of Wnt proteins (38). However, it is likely that multiple PORCN Cys residues are palmitoylated similar to the situation with HHAT because mutation of Cys-187 to an Ala did not completely abrogate PORCN palmitoylation (38). Furthermore, it is unclear how a primate-specific Cys in PORCN would modulate Wnt activity so radically, especially because the function of PORCN is completely conserved in mice, which do not have this Cys residue. Unlike PORCN, all Cys residues palmitoylated in HHAT are conserved in primates and other mammalian sequences, such as mice and rat (supplemental Fig. S1). Significantly, mutation of the His residue of PORCN did not affect palmitoylation of the protein, suggesting that this is a unique feature for HHAT. This result, when taken into consideration with the data that show that HHAT is the only known MBOAT in which mutation of the His residue still retains some activity of the protein, may suggest that HHAT does function in an unique manner compared with other MBOAT proteins.

Unlike the *N*-palmitoylation of Hh proteins, *S*-palmitoylation is catalyzed by the DHHC PATs and attaches palmitic acid to proteins via thioester linkages to cysteine residues. This is a reversible process via the function of the acyl protein thioesterases, and, as such, it can be considered as an “on-off” switch similar to protein phosphorylation (39). Palmitoylation of membrane proteins has been shown to affect association with other proteins (40) or the targeting of ER membrane proteins to specific domains within the ER membrane (41). HHAT palmitoylation could potentially have a similar effect on the protein. Future studies, perhaps with mass spectrometry analysis of HHAT-associated proteins or superresolution microscopy of HHAT mutants, should provide clues to both possibilities. Insertion of the palmitates into the cytoplasmic leaflet of the ER may also locally affect membrane curvature to aid in the interaction with the acyl-CoA or Hh substrates.

Another potential function is that palmitoylation affects the structure of HHAT. Palmitoylation of model proteins with a single TM in residues adjacent to the TM disrupts the orientation of the helices within a lipid bilayer (42). Palmitoylation of HHAT may affect the tilting of the TMs within the ER membrane, therefore affecting protein function. Repeating the experiments presented in this study with palmitoylation-deficient mutants of HHAT and seeing whether topology is altered could test this hypothesis.

The result that the H379A mutant is not palmitoylated would perhaps suggest that the protein is actually autoacylated. However, because of the fact that the D339N HHAT mutant is only weakly active and yet still fully palmitoylated, it is likely that HHAT palmitoylation is not caused by autoacylation of the protein. Therefore, identification of the DHHC PAT responsible for HHAT palmitoylation and the interplay with the acyl protein thioesterases may provide a further dimension of how this protein is regulated by palmitoylation.

Finally, during mPEG accessibility analysis on the HHAT Cys mutants, an unexpected result was seen with the C324A mutant. The mPEG labeling produced significantly more bands with a laddering effect of differentially tagged HHAT proteins (Fig. 6*C*). The increase in molecular weight could be attributed to increased multimerization of the protein. It is still unclear whether MBOAT family proteins form functional multimers within the cell, although studies on GOAT suggest that the protein functions as a monomer (21). Intriguingly, this result could further suggest that the protein conformation is significantly altered in this protein because more Cys residues within the protein are more accessible. The importance of this Cys residue is emphasized by the facts that it is the only Cys residue completely conserved in all examined species (supplemental Fig. S1) and that it is also palmitoylated (Fig. 7). Further studies with this mutant should reveal more as to its importance in regulating HHAT function.

In summary, we provide a comprehensive analysis of HHAT topology, showing that the protein consists of ten TMs and two RLs, with the His residue of the proposed catalytic site facing the lumen of the ER. We provide evidence that HHAT is palmitoylated on multiple Cys residues on the cytoplasmic side of the protein and that mutation of the invariant His residue of the MBOAT domain affects palmitoylation of HHAT while also showing that Cys-324 of the protein may be important for controlling its folding. From this study, it is clear that HHAT is an extremely complex protein with multiple factors affecting its function. This study provides the foundation on which to build an understanding to how HHAT functions and how it may be inhibited and creates new avenues of inquiry for future studies of this important class of proteins.

## Supplementary Material

Supplemental Data

## Footnotes

8 T. Lanyon-Hogg, A. I. Magee, and E. W. Tate, manuscript in preparation.

## Footnotes

7 The abbreviations used are:

Hh: Hedgehog
PAT: protein acyltransferase
HHAT: Hedgehog acyltransferase
TM: transmembrane domain
ER: endoplasmic reticulum
MBOAT: membrane-bound *O-*acyltransferase
PORCN: Porcupine
GOAT: ghrelin-*O*-acyltransferase
RL: re-entrant loop
YnPalm: alkynyl-palmitate analog pentadec-14-ynoic acid
CuAAC: copper-catalyzed [3 + 2] cycloaddition
AzTB: azido-TAMRA-PEG-Biotin
mPEG: maleimide-PEG
TEV: tobacco etch virus
DMSO: dimethyl sulfoxide
TAMRA: carboxytetramethylrhodamine
TCEP: Tris(2-carboxyethyl)phosphine
TBTA: Tris[(1-benzyl-1*H*-1,2,3-triazol-4-yl)methyl]amine.
