# Supplementary material for: Topological Analysis of Hedgehog Acyltransferase, a Multipalmitoylated Transmembrane Protein
Source: J Biol Chem. 2014 Dec 12;290(6):3293–307. doi: 10.1074/jbc.M114.614578 (PMC4319003; doi:10.1074/jbc.M114.614578)
Supplement: Supplemental Data [file supp_290_6_3293__index.html]

Topological analysis of Hedgehog acyltransferase, a multi-palmitoylated transmembrane protein — Topological Analysis of Hedgehog Acyltransferase, a Multipalmitoylated Transmembrane Protein — Topological Analysis of HHAT — Supplemental Data 

# Topological Analysis of Hedgehog Acyltransferase, a Multipalmitoylated Transmembrane Protein

## Supplemental Data

**Files in this Data Supplement:**

- Sup Figure 1 new (.pdf, 3.6 MB) - Supplementary Figure 1
