# Supplementary material for: Topological Analysis of Hedgehog Acyltransferase, a Multipalmitoylated Transmembrane Protein
Source: J Biol Chem. 2014 Dec 12;290(6):3293–307. doi: 10.1074/jbc.M114.614578 (PMC4319003; doi:10.1074/jbc.M114.614578)
Supplement: Supplemental Data [file supp_M114.614578_jbc.M114.614578-1.pdf]

```
H.sapiens               -----------------------------------------------------------M  1
M.fascicularis          -----------------------------------------------------------M  1
P.troglodytes           -----------------------------------------------------------M  1
P.paniscus              -----------------------------------------------------------M  1
N.leucogenys            -----------------------------------------------------------M  1
M.musculus              -----------------------------------------------------------M  1
R.norvegicus            -----------------------------------------------------------M  1
S.scrofa                -----------------------------------------------------------M  1
C.hircus                -----------------------------------------------------------M  1
C.lupus                 -----------------------------------------------------------M  1
M.putorius              MFTPQAVKQPVRKARLPSTPPSYLLPPAERRGDHWAGFRAPWSFRNSQCVCLDPSSQGTM  60
O.orca                  -----------------------------------------------------------M  1
C.simum                 -----------------------------------------------------------M  1
D.rerio                 ---------------------------------------------------MTPKWELVS  9
A.mississippiensis      ----------------------------------------------------------MV  2
V.pacos                 -----------------------------------------------------------M  1
C.ferus                 -----------------------------------------------------------M  1
P.sinensis              ------------------------------------------------------MLQVAM  6
M.lucifugus             -----------------------------------------------------------M  1
L.chalumnae             ------------------------------------------MQNSGNQKPQKPALRIVR  18
P.hodgsonii             -----------------------------------------------------------M  1
B.mutus                 -----------------------------------------------------------M  1
C.elegans               -----------------MTPSHPEVPPPRQKQ-----------------------TLFEY  20


H.sapiens               LPRWELALYLLASLGFHFYSFYEVYKVSREHEEELDQEF----ELETDTLFGGLKKDATD  57
M.fascicularis          LPRWELALYLLASLGFHFYSFYEVYKVSREHEEELDQEF----ELETDTLFGGLKKDATD  57
P.troglodytes           LPQWELALYLLASLGFHFYSFYEVYKVSREHEEELDQEF----ELETDTLFGGLKKDATD  57
P.paniscus              LPQWELALYLLASLGFHFYSFYEVYKVSREHEEELDQEF----ELETDTLFGGLKKDATD  57
N.leucogenys            LPRWELALYLLASLGFHFYSFYEVYKVSREHEEELDQEF----ELETDTLFGGLKKDATD  57
M.musculus              LPGWELTLCLLVSLGFHFRSFYEVYKVSREHEEELDQEF----ELEMDTLFGGLKKDPTD  57
R.norvegicus            LSRWELTLCLLVSLGFHFRSFYEVYKVSREHEEELDQEF----ELEMDTLFGGLK-----  52
S.scrofa                LPRWELSFYLFASLGFHFYSFYEVFKVSREHEEELDQEF----DLETDSLFGGLKKDPTD  57
C.hircus                LPWWELSFYLLASIGFHFYSFYEVYKVSREHEEELDREF----DLETGTLFGGLKKDPTD  57
C.lupus                 LPWWELSFYLCASLGFHLYSFYEVYKASREHEEELDRQF----DLEIGTLFGGLKKDPND  57
M.putorius              LPRWELSFYLLASLGFHLYSFYEVYKASREHEEELDRQF----ALEIGTLFGGLKKDPTD  116
O.orca                  LPRWELSFYLFASLGFHFYSFYEVYKVSREHEEELDQEF----DLDTDTLFGGLKKDPTD  57
C.simum                 LPRWELSLYLLASLGFHFYSFYEVYKVSREHEEELDREF----DLETDTLFGGLKKDPTD  57
D.rerio                 LPRWEMVAYWILSFSSHFYSFYQLHSFSKEHEVSLDKEV----QLERGFLLWGFKKDPTD  65
A.mississippiensis      LPLWEMCLYLFLSFGFHFYSFYEVYKVSREYEEELDREF----ELEKNALFWGLKKDPTD  58
V.pacos                 LPRWELFLYLLTSLGFHFYSFYEVYKTSREHEEELDQEF----DLETDTLFGGLKKDPTD  57
C.ferus                 LPRWELFLYLLTSLGFHFYSFYEVYKTSREHEEELDQEF----DLETDTLFGGLKKDPTD  57
P.sinensis              LPLWEMFLYLLLSFGFHFYSFYEVYKVSREYEEELEREF----ELEKDTLFWGLKKDPTD  62
M.lucifugus             LPRWELSLYLLASLGFHFYSFYEVYKTSREHEEQLDQEF----DLAIGTLLGGFKKDQTD  57
L.chalumnae             LPRWEIGIYLVLSFGSHLYSFYEVHKISRDYEDDLDSEF----ELEKGFFIWGFKKDPTD  74
P.hodgsonii             LPWWELSFYLLASIGFHFYSFYEVYKVSREHEEELDREF----DLETGTLFGGLKKDPTD  57
B.mutus                 LPWWELSLYLLASIGFHFYSFYEVYKVSREHEEELDREF----DLETGTLFRGLKKDPTD  57
C.elegans               PPLKEVPICFAVTVCTMAYAWYGVYRASQQFEWSIGDYGIWSSPPIIGNLTGPLMKDVSN  80
                            *:      :.     ::* :.  *::.* .:              :   :

H.sapiens               FEWSFWMEWGKQWLVWLLLGHMVVSQMATLLARKHRPWILMLYGMWAC---WCVLGTPGV  114
M.fascicularis          FEWSFWMEWGKQWLVWLLLGHMVVSQMATLLARKHRPWILMLYGMWAC---WCVLGTPGV  114
P.troglodytes           FEWSFWMEWGKQWLVWLLLGHMVVSQMATLLARKHRPWILMLYGMWAC---WCVLGTPGV  114
P.paniscus              FEWSFWMEWGKQWLVWLLLGHMVVSQMATLLARKHRPWILMLYGMWAC---WCVLGTPGV  114
N.leucogenys            FEWSFWMEWGKQWLVWLLLGHMVVSQMATLLARKHRPWILMLYGMWAC---WCVLGTPGV  114
M.musculus              FEWNFWMEWGKRRLVWLFIGHMAVSQLATLLTKKHRPWIVMVYGMWAC---WCVLGAPGV  114
R.norvegicus            ---------------------------------KHRPWIIMIYGMWAC---WCVLGAPGV  76
S.scrofa                FEWSFWMEWGKQRLAWLLLGHVGVSQVANLLARKHRPWILAAYGMWAC---HCALGTRGT  114
C.hircus                FEWSFWMEWGKERLVWLFLGHMSVSQVANLLSRKHKPWILAAYGMWAC---RCVLGTRGT  114
C.lupus                 FEWSFWMEWGKQRLVRFLFGHMVVSQMANVLARKHRPWILGAYGMWAS---WCVLGAHGT  114
M.putorius              FEWSFWMEWGKQRLLRFLFGHVAVSQLANVLARKHRPWILGAYGIWAS---WCVLGAHGT  173
O.orca                  FEWSFWMEWGKQRLVWLLLGHMGVSQVANLLTRKHKPWILTAYGMWAC---WCVLETQGT  114
C.simum                 FEWSFWMEWGKQWLVWLLLGHAAVSQMANLLARKHRPWILTAYGMWAC---WCVLGAPGV  114
D.rerio                 FEWSFWNEWAWRSLLWSLIGHAVVSRLAYYYLPQYRLAVFAMYGLISA---GWILGVRGV  122
A.mississippiensis      FEWSFWMEWGKGCILWLLFGHLAVSQISRLFLEKYKPWCLMTYGMIAC---WFLLGIKGL  115
V.pacos                 FEWNFWMEWGKQRLAWLLLGHVGVSQVANLLSRKHRPWILAAYGMWAC---QCVLGAQGT  114
C.ferus                 FEWNFWMEWGKQRLAWLLLGHVGVSQVANMLTRKHRPWILAAYGMWAC---QCVLGAQGT  114
P.sinensis              FEWTFWMEWGKERIIWLLLGHLMVSQMSRLFMEKYQPWCLMAYGMVAC---WFLLGINGL  119
M.lucifugus             FEWSFWMEWGKQRLVWLLLGHLAVSQMANQIARKHRPWFLTAYGMWAC---WCVLGTPGT  114
L.chalumnae             FEWSFWTEWGKRSLLWTLLGHVIISQVTSVVISELRLWFLMAYGMMAC---LFVLGLKGL  131
P.hodgsonii             FEWSFWMEWGKERLVWLFLGHMSVSQVANLLSRKHKPWILAAYGMWAC---SCVLGTRGT  114
B.mutus                 FEWSFWMEWGKERLVWLFLGHMSVSQVANLISRKHKPWILAVYGMWAC---RCVLGTQGT  114
C.elegans               WEWSRWSPFALSYL-PVFFGHFLVFNIGSAILPESAF--IPIYTLCSIAAVAYNFTPFLV  137
                                                         :     .  * : :       :
```

Figure S1

```
H.sapiens             AMVLLHTTISFCVAQF-RSQLLTWLCSLLLLSTLRLQGVEEVKRRWYKTENEYYLLQFTL  173
M.fascicularis        AMVLLHTTISFCVAQF-RSQLLSWLCSLLLLSTLRLQGVEEVKRRWYKTENEYYLLQFTL  173
P.troglodytes         AMVLLHTTISFCVAQF-RSQLLTWLCSLLLLSTLRLQGVEEVKRRWYKTENEYYLLQFTL  173
P.paniscus            AMVLLHTTISFCVAQF-RSQLLTWLCSLLLLSTLRLQGVEEVKRRWYKTENEYYLLQFTL  173
N.leucogenys          AMVLLHTTISFCVAQF-RSQLLTWLCSLLLLSTLRLQGVEEVKRRWYKTENEYYLLQFTL  173
M.musculus            VMVLLHSTIAFCVAQF-RSVLLSWLCSLLLLSTLRLQSVEEVKRRWYKTENEYYLLQFTL  173
R.norvegicus          VMVLLHTTIAFCVAQF-RSMLLSWLCSLLLLSTLRLQSVEEVKRRWYKTESEYYLLQFTL  135
S.scrofa              AMVLLHTTISFCVAQF-RSLALSWLCSLFLLSTLRLQDVEEVKRGWYETENEYYLLQFTL  173
C.hircus              AMVLIHTTVSFCVAQF-RSQFLSWLCSLSLLSTLRLQDVEEVKRGWCTTENECYLLQFTL  173
C.lupus               AIIFLHTLISFCVAQL-RSLALTWLCSLFLLSTLRLQDVEEVKRGWYQTENEYYLLQFTL  173
M.putorius            AIIFLHTLISFCVAQF-RSLFLTWLCSLLLLSTLRLQDVEEVKRGWYQTENEYYLLQFTL  232
O.orca                AMVLIHTTISFCVAQF-RSLFLSWLCSLFLLSTLRLQDVEEVMRGWYETENEYYLLQFTL  173
C.simum               AMVFLHTIISFCVAQF-RSLLLSWLCSLLLLSTLRLQDMEEVKRGWYQTENEYYLLQFTL  173
D.rerio               IVLMLHLSISLVVAQL-RSPVLSWFCSLLLLSTLNVSALQDLQRGWYTSEDQYYLLLFST  181
A.mississippiensis    ALILLHIFISFSVAQF-QMSILTWLCSLLLLSTLRIPALEEAKRGWYDTENEYYLLLFTL  174
V.pacos               AVVLLHTAISYCVAQF-RSPFLSWLCSLLLLSTLRLQDVEEVKRGWYQTENEYYLLQFTL  173
C.ferus               AVVLLHTAISYCVAQF-RSLFLSWLCSLLLLSTLRLQDVEEVKRGWYQTENEYYLLQFTL  173
P.sinensis            AVLLLHIAISFSVAQF-QTAVLTWLCSLLLLSALRVPALEEAMRGWYDSENEYYLLLFTL  178
M.lucifugus           AMIFLHTVISFCVAQF-RSLFLSWLCSLLLLSTLRLQDVEEIKRGWYKTENEYYLLQFTL  173
L.chalumnae           VVILLHTLASFTVAQL-RRPLLSWLCSLMLLYTLHMSSLEEIQRDWYETENEYYLLLFSL  190
P.hodgsonii           AMVLIHTTISFSVAQF-RSQFLSWLCSLSLLSTLRLQDVEEVKRGWCTTENECYLLQFTL  173
B.mutus               AMVLIHATISFCVAQF-RSQFLSWLCSLSLLSTLRLQDVEEVKRGWYKTENEYYLLQFTL  173
C.elegans             AVSMAQGTVVFLTAMYAKKTWAVWLSALPVLYV---SMHE-----TNSLSDDPFLIFTFL  189
                       : : :      .*   :     *:.:* :* .      :         ..: :*:

H.sapiens             TVRCLYYTSFSLELCWQQLPAA--------------STSYSFPWMLAYVFYYPVLHNGPI  219
M.fascicularis        TVRCLYYTSFSLELCWQQLPAA--------------STSYSFPWMLAYVFYYPVFHNGPI  219
P.troglodytes         TVRCLYYTSFSLELCWQQLPAA--------------STSYSFPWMLAYVFYYPVLHNGPI  219
P.paniscus            TVRCLYYTSFSLELCWQQLPAA--------------STSYSFPWMLAYVFYYPVLHNGPI  219
N.leucogenys          TVRCLYYTSFSLELCWQQLPAA--------------STSYSFPWMLAYVFYYPVLHNGPI  219
M.musculus            TVRCLYYTSFSLELCRQPPSAQPTPSA--------QGASHSYPWLLTYVFYYPVFHNGPI  225
R.norvegicus          TVRCLYYTSFSLELCRQPPSAQ--------------RACFSFPWLLTYVFYYPVFHNGPI  181
S.scrofa              TVRCLYYTSFSLELCWQQLPAE--------------RAFYSFPWMVAYVFYYPVFHNGPI  219
C.hircus              AVRCLCYTSFSLELCWRPPPAE--------------RPSYSLPWMLAYVFYYPVFHNGPV  219
C.lupus               TVRCLYYTSFSLEFCWQQMPNG--------------HSFYSFPWLVAYVFYYPVFHNGPI  219
M.putorius            TVRCLYYTSFSLEFCWQEVPCG--------------SSFYSFPWLVAYVFYYPVFHNGPI  278
O.orca                TVRCLYYTSFSLELCWQQPPAE--------------RTFCSFPWMVAYVFYYPVFHNGPI  219
C.simum               TVRCLYYTSFSLEFCWQQRPAG--------------RTSYSFPWLVAYVFYYPVFHNGPI  219
D.rerio               AVCSLHCISFCLEMCWHPLQAGRPKHCSSLQNCALKTLIYQFLKLTAFCFYHPLFYNGPI  241
A.mississippiensis    TVRCIYYTSFSLEYCWHGST---------------QKRYHSFLWMLAYMFYYPVFHNGPI  219
V.pacos               TVRCLYYTSFSLEFCWQPRPAG--------------HTFYSFPWMVAYVFYYPVFHNGPV  219
C.ferus               TVRCLYYTSFSLEFCWQPRPAG--------------HTFYSFPWMVAYVFYYPVFHNGPI  219
P.sinensis            TVRCLYYTSFSLEYCWHGST---------------QKRYHSFFWMLAYMFYYPVFHNGPI  223
M.lucifugus           TVRCLYYTSFSLEFCWQQLPCG--------------RTFYSLPWMMAYVFYYPVFHNGPI  219
L.chalumnae           AICSLRYTSFSLEHCWYQSS---------------EKKYKLFLWMLAYLFYYPVFHNGPV  235
P.hodgsonii           AVRCLCYTSFSLELCWRPPPAE--------------RPSYSLPWMLAYVFYYPVFHNGPV  219
B.mutus               TVRCLYYTSFSLELCWQPPPAE--------------RPFYSLPWMLAYVFYYPVFHNGPV  219
C.elegans             SYSMIHYISYCLEHLKGESRKE----D--------DTILKRYLRMMFFAFYQPYLF-SLI  236
                      :   :   *:.**                               :  : ** * :. . :

H.sapiens             LSFSEFIKQMQQQEHDSLKASLCVLALGLGRLLCWWWLAELMAHLMYMHAIYSSIPLLET  279
M.fascicularis        LSFPEFIKQMQQQEHESLKGNLCVLALGLGRLLCWWWLAELMAHLMYMHAIYSSIPLLEA  279
P.troglodytes         LSFSEFIKQMQQQEHDSLKASLCVLALGLGRLLCWWWLAELMAHLMYMHAIYSSIPLLET  279
P.paniscus            LSFSEFIKQMQQQEHDSLKASLCVLALGLGRLLCWWWLAELMAHLMYMHAIYSSIPLLET  279
N.leucogenys          LSFPDFIKQMQQQQHDSLKASLCVLALGLGRLLCWWWLAELMAHLMYVHAIYSSIPLLET  279
M.musculus            LNFPEFFRQMQQPELNSLQHSLCIVAKGLGRLLCWWWLAELMVHLMYMHALYSSAPLLES  285
R.norvegicus          LNFPEFFRQMQQPELSSLQHSLCILAKGLGRLLCWWWLAELMVHLMYMHALYSSIPLLES  241
S.scrofa              LSFPEFLTQMQPREQGSLKASFSILVRGLGRLLFYWWLVELMVHLMYMHAICSSAPLLSA  279
C.hircus              LSFPEFIAQMQQQEQSSPKTSLSILALGLGRLLFYWWLAELMVHLMYMHAIYSSASLLRA  279
C.lupus               LSFPEFIAKVQQQEYCSLKSNLSAFAVGLGRLLCYWWLAELMVHLMYMHAIYSSASLLKA  279
M.putorius            LSFPEFIAKMQQQEYCSLKSNLSAFAVGLGRLLCYWWLAELMVHLMYMHAIYSSASLLRA  338
O.orca                LSFPEFIAQMQQQEQGSPKAGLPTLARGLGRLLSCWWLAELMVHLMYMHAIYSSTSLLRA  279
C.simum               LSLPEFIRQMQQQERRSMKASLSVLVLGLGRLLCSWWLAELMVHLMYMHAIYSSTPLLGA  279
D.rerio               MTYKDFSEQIERPLCK---VSALHVFSGILRLCVWWCLAEFMIHFMYMHAIQSNETYLEM  298
A.mississippiensis    MNFDEFSKQMRRQEACRLKTNFRILILGMVRIFFWWWLAELMIHLMYMHAFYSSSPHLEA  279
V.pacos               LSFPEFIGQMQQQEHGSLKASFSILVLGLGRLLFCWWLAELMVHLMYMHAIYSSTSLLRA  279
C.ferus               LSFPEFIGQMQQQEHGSLKASFSILVLGLGRLLFCWWLAELMVHLMYMHAIYSSTSLLRA  279
P.sinensis            MTFNEFTKQMRRQEACSLKSNLCVLLLGVVRIFFWWWLAELIIHLMYIHAIYSSSSHLEA  283
M.lucifugus           LSFPEFIGQMRQPEQCPLRLSLPVLARGLGRLFVCWCLAELMVHLMYMHAFYGSAPLLRA  279
L.chalumnae           ISYDEFSKQIQKQQTRVSKMEVYALTLSICRIFLWWWLAESMIHLMYIHAIPSSNTVLDA  295
P.hodgsonii           LSFPEFIAQMQQQEQSSPKTSLSILALGLGRLLFYWWLAELMVHLMYMHAIYSSASLLRA  279
B.mutus               LSFPEFIAQMQQQEQSSPKTSLSILALGLGRLLFYWWLAELMVHLMYMHAIYSSASLLRA  279
C.elegans             VLYPDFERQLRERKTRQ--RNWLDCAWSGVRIAFWWWLMEASLHFLYHEAILKNAPYLYS  294
                      :   :*  ::.                .  *:   * * *   *::* .*:  .   *
```

```
H.sapiens               VSCWTLGGLALAQVLFFYVKYLVLFGVPALLMRLDGLTPPALPRCVSTMFSFTGMWRYFD 339
M.fascicularis          VSGWTLGGLALAQVLFFYVKYLVLFGVPALLMRLDGLTPPALPRCVSTMFSFTGMWRYFD 339
P.troglodytes           VSCWTLGGLALAQVLFFYVKYLVLFGVPALLMRLDGLTPPALPRCVSTMFSFTGMWRYFD 339
P.paniscus              VSCWTLGGLALAQVLFFYVKYLVLFGVPALLMRLDGLTPPALPRCVSTMFSFTRMWRYFD 339
N.leucogenys            VSCWTLGGLALAQVLFFYVKYLLLFGVPALLMRLDGLTPPALPRCVSTMFSFTGMWRYFD 339
M.musculus              VSCWTLGGLALAQVLFFYVKYLVLFGVPALLMRLDGLTPPPLPRCVSTMFSFTGMWRYFD 345
R.norvegicus            VSCWTLGGLALAQVLFFYVKYLVLFGAPALLMRLDGLTPPPLPRCVSTMFSFTGMWRYFD 301
S.scrofa                VSCWTLGGLALAQVLFFYVKYLVLFGVPALLMRLDGLSPPPLPRCVSTMFSFTGMWRYFD 339
C.hircus                VSSWTLGGLALAQVLFFYVKYLVLFGVPALLMRLDGLRPPPLPRCVSTMFSFTGMWRHFD 339
C.lupus                 VSCWTLGGLALAHVLFFYLKYLVLFGVPALLMRLDGLTPPPLPRCVSTMFSFTGMWRYFD 339
M.putorius              VSCWTLGGLALAHVLFFYLKYLVLFGVPALLMRLDGLTPPPLPRCVSTMFSFTGMWRYFD 398
O.orca                  VSCWTLGGLALAQVLFFYVKYLVLFGVPALLMRLDGLTPPPLPRCVSTMFSFTGMWRYFD 339
C.simum                 VSSWTLGGLALAQVLFFYVKYLVLFGVPALLMRLDGLTPPPLPRCVSTMFSFTGMWRYFD 339
D.rerio                 LPPWAMGGLALALVQFFYVKYLVLFGAVSLLVRLDGLEPPTLPRCVSIMYSFTGMWRHFD 358
A.mississippiensis      MSDWTLGGLALAQVLFFYVKYLVLFGVPALIIQMDGLKPPALPRCVSTMHSFTGMWRSFD 339
V.pacos                 VSCWTLGGLALAQVLFFYVKYLVLFGVPALLMRLDGLTPPPLPRCVSTMFSFTGMWRYFD 339
C.ferus                 VSCWTLGGLALAQVLFFYVKYLVLFGVPALLMRLDGLTPPPLPRCVSTMFSFTGMWRYFD 339
P.sinensis              VSYWTLGGLALAQVLFFYVKYLVLFGIPALIIRMDGLKPPALPRCVSTMHSFAGMWRSFD 343
M.lucifugus             VSCWTLGGLALAQVLFFYVKYLVLFGIPALLMRLDGLTPPPLPRCVSTMFSFTGMWRYFD 339
L.chalumnae             VESWALGGLALAQVLFFYVKYLVLFGIPNLVVRMDGIEPPRLPRCVSTMYSFTGMWRHFD 355
P.hodgsonii             VSSWTLGGLALAQVLFFYVKYLVLFGVPALLMRLDGLRPPPLPRCVSTMFSFTGMWRHFD 339
B.mutus                 VSSWTLGGLALAQVLFFYVKYLVLFGVPALLMRLDGLRPPPLPRCVSTMFSFTGMWRHFD 339
C.elegans               LPKDQFVALGMAMGIFFHLKYAVIFGLPTLFAKLDNMDPLPGPICLIRVTLYSKVWREFD 354
                        :    : .*.:*   **::** ::**   *. ::* : *   * *:  :  :: :** **

H.sapiens               VGLHNFLIRYVYIPVGGSQHGLLGTLFSTAMTFAFVSYWHGGYDYLWCWAALNWLGVTVE 399
M.fascicularis          VGLHKFLIRYVYIPVGGSQHGLLGTLFSTAMTFAFVSYWHGGYDYLWCWAALNWLGVTVE 399
P.troglodytes           VGLHNFLIRYVYIPVGGSQHGLLGTLFSTAMTFAFVSYWHGGYDYLWCWAALNWLGVTVE 399
P.paniscus              VGLHNFLIRYVYIPVGGSQHGLLGTLFSTAMTFAFVSYWHGGYDYLWCWAALNWLGVTVE 399
N.leucogenys            VGLHNFLIRYVYIPVGGSQHGLLGTLLSTAMTFAFVSYWHGGYDYLWCWAALNWLGVTVE 399
M.musculus              VGLHNFLIRYVYIPLGGSQHGLLGTLLSTATTFAFVSYWHGSYEDLWCWAALNWLGVTVE 405
R.norvegicus            VGLHNFLIRYVYIPLGGSQHGLLGTLLSTAMTFAFVSYWHGSYEDLWCWAALNWLGVTVE 361
S.scrofa                VGLHDFLVRYVYIPVGGSQHGLLGTLFSTAVTFAFVSYWHGGNDYLWCWAALNWLGVTVE 399
C.hircus                VGLHDFLVRYVYIPAGGSQHGLLGALFSTAMTFAFVSFWHGGNDYLWCWATLNWLGVTVE 399
C.lupus                 VGLHDFLIRYVYIPVGGSQHGLLGTLFSTAVTFAFVSFWHGGYDYLWCWAALNWLGVTVE 399
M.putorius              VGLHDFLIRYVYIPVGGSQHGLLGTLFSTAVTFAFVSFWHGGYDYLWCWAALNWLGVTVE 458
O.orca                  VGLHDFLVRYVYIPAGGSQHGLLGTLFSTAMTFAFVSFWHGGNDYLWCWATLNWLGVTVE 399
C.simum                 VGLHNFLIRYVYIPVGGSQHGLLGTLFSTALTFAFVSYWHGGYDYLWCWAALNWLGVTVE 399
D.rerio                 VGLYKWLIRYIYVPLGGSRHGAFRKLLSTALAFGFVCFWHGCHDYLLYWALLNWVGVLVE 418
A.mississippiensis      VGLHHFLIRYIYIPMGGSQCSVVGMLFSTAITFAFVSYWHGGHNYLWYWAALNWLGVTAE 399
V.pacos                 VGLHDFLIRYVYIPMGGSQHGLLGTLFSTAMTFAFVSFWHGGNDYLWCWAALNWLGVTVE 399
C.ferus                 VGLHDFLIRYVYIPMGGSQHGLLGTLFSTAMTFAFVSFWHGGNDYLWCWAALNWLGVTVE 399
P.sinensis              VGLHHFLIRYIYIPMGGSHGGVFGMLFSTAATFSFVSYWHGGHNYLWFWAALNWLGVIAE 403
M.lucifugus             VGLHDFLIRYVYIPVGGSQHGLLGTLFSTAMTFAFVSYWHGGHDYLWCWAALNWLGVTVE 399
L.chalumnae             VGLHRWLIRYIYIPMGGSQHGSLGMMLSTAMAFGFVYYWHGGHNYLFNWAVLNWLGVILE 415
P.hodgsonii             VGLHDFLVRYVYIPAGGSQHGLLGALFSTAMTFAFVSFWHGGNDYLWCWATLNWLGVTVE 399
B.mutus                 VGLHDFLVRYVYIPAGGSQHGLLGALFSTAMTFAFVSFWHGGNDYLWCWATLNWLGVTVE 399
C.elegans               RGLYQFFKTYIFIPICAPTFSLPRKIFGVFISYGFVLLWHGFYHHNIVWIGLNILALFIE 414
                          **: ::   *:::*  .    .      ::..  ::.**  ***   .    *  ** :.:  *

H.sapiens               NGVRRLVETPCIQDSLARYFSPQARRRFHAALASCSTSMLILSNLVFLGGNEVGKTYWNR 459
M.fascicularis          NGVRRLVEIPCIQDSLTRYFSPQARHRFHAALASCSTSMLILSNLVFLGGNEVGKTYWNR 459
P.troglodytes           NGVWRLVETPCIQDSLARYFSPQARRRFHAALASCSTSMLILSNLVFLGGNEVGKTYWNR 459
P.paniscus              NGVWRLVETPCIQDSLARYFSPQARRRFHAALASCSTSMLILSNLVFLGGNEVGKTYWNR 459
N.leucogenys            NGVRRLVETPCMQDSLARYFSPQARRRFHAAFASCSTSMLILSNLVFLGGNEVGKTYWNR 459
M.musculus              SGVRRLLETPCVRETLARHLSPQAHHRLHALLAACSTSMLILFNLVFLGGIQVGKTYWNR 465
R.norvegicus            SGVRRLLQTPCVRETLARHLSPQARRRLHALLASCSTSMLILFNLVFLEGIQVGKTYWNR 421
S.scrofa                SGVRRLVQKPSIQDSLAQVLSPQARRRFHAALASCSTSMLILSNLVFLGGNQVGRIYWNR 459
C.hircus                NGVQKLVQRPRVQHSLAQFLSPGARRRLHAALASCSTSMLILSNLVFLGGSQVGEIYWDR 459
C.lupus                 NVVQRLVRTPCIQDSVIQFLSPQARRRFHAALASCSTSMLILSNLVFLGGSQVGKIYWNR 459
M.putorius              NAVQRLVQTPYVRDSVVQFLSPQARRRFHAALASCSTAMLILSNLVFLGGSQVGKIYWNR 518
O.orca                  NGVQRLVQAPRVQDSLAQFLSPQARRRLHAALASCSTSMLILSNLVFLGGNQVGKIYWNR 459
C.simum                 NGVRRLVETPCIRDSLARFLSPQARRRFHAALASCSTSILILSNLVFLGGNQVGKIYWNR 459
D.rerio                 NAFALLFSSPPLRHAIVYCLTPRMQRRGLALISAFSTAFLILSNLFFLGGTHVGRIFWKK 478
A.mississippiensis      NGVKRLLSVSLVRDLIDHFFLPRICRRLHAVLAAVSTSMLILSNLVFLGGNDVGKIYWDW 459
V.pacos                 NGVQRLVQTPRIQDSLAQLLSPPARRRFHAALASCSTSMLILSNLVFLGGNHVGKIYWTR 459
C.ferus                 NGVQRLVQTPRVQNSLAQLLSPPARRRFHAALASCSTSMLILSNLVFLGGNHVGKIYWTR 459
P.sinensis              NGVRRLLSVSLVQELIDRFFSPGICRRLHAALAAISTSMLILSNLVFLGGNQVGKIYWNR 463
M.lucifugus             NGVRRLVETPCIQDSLVQFLSPRARRRFHAALASCSTSMLILSNLVFLGGNQVGKIYWNR 459
L.chalumnae             NGVKKVLSIPSIQNLIELSFSAEMQRRGHAALAAFSTAMLILSNLIFLGGNQVGTIYWNR 475
P.hodgsonii             NGVQKLVQRPHVQHSLAQFLSPGARRRLHAALASCSTSMLILSNLVFLGGSQVGEIYWDR 459
B.mutus                 NGVQKLVQRPHVQHSLAQFLSPGARRRLHAALASCSTSMLILSNLVFLGGSQVGEIYWDR 459
C.elegans               MGSKAIYTIESVRKWREANLSDVAFRRILAWCHILPFAIGLYSNFYFLGGSDVGAAFVQR 474
                            :     ::.     :     :*  *        :: :  *: ** * .**  :
```

```
H.sapiens              IFIQG-----WPWVTLSV-------------LGFLYCYSHVGIAWAQTYATD--------  493
M.fascicularis         IFIQG-----WPWVTLPV-------------LGFLYCYSHVGIAWAQTYAMD--------  493
P.troglodytes          IFIQG-----WPWVTLSV-------------LGFLYCYSHVGIAWAQTYATD--------  493
P.paniscus             IFIQG-----WPWVTLSV-------------LGFLYCYSHVGIAWAQTYATD--------  493
N.leucogenys           IFIQG-----WPWVTLSV-------------LGFLYCYSHVGIAWAQTYAMD--------  493
M.musculus             IFLQG-----WPWVTLSV-------------LGFLYCYSHVDIAWAQTYTVL--------  499
R.norvegicus           IFLQG-----WPWVTLSV-------------LGFLYCYSHVGIAWAQTYTVL--------  455
S.scrofa               IFIQG-----WPWVTLSV-------------LGFLYCYSHVGIAWAQTYAVD--------  493
C.hircus               IFIQG-----WPCVTLSV-------------LGCLYCYAHVGIAWAHTYSGN--------  493
C.lupus                IFIQG-----ADGLPGPPSPARAPGRAATTTTGAWFYYPSVLC-----------------  497
M.putorius             IFVQG-----WPYVTLSV-------------LGFLYCYSHVGIAWAQTYSVD--------  552
O.orca                 IFIQG-----WPCVTLSV-------------LGFLYCYSHVGIAWAQTYSVN--------  493
C.simum                IFIQG-----WPWVTLSV-------------LGFLYCYSHVGIAWAQTYSMD--------  493
D.rerio                LFVQG-----LLESSLEDLCVAG-------------------------------------  496
A.mississippiensis     IFVKG-----WPWATLSV-------------IGFLYCYSQVGIEWDQMYIAD--------  493
V.pacos                IFIQG-----WPWVTLSV-------------LGFLYCYSHVGIAWAQTYS----------  491
C.ferus                IFIQG-----WPWVTLSV-------------LGFLYCYSHVGIAWAQTYS----------  491
P.sinensis             IFVEG-----WPWATLSV-------------FGFLYCYSHVGIEWDHTHCAD--------  497
M.lucifugus            IFIQG-----WPWVTLSV-------------LGFLYCYSHVGIAWAQTYSMN--------  493
L.chalumnae            IFVQG-----QNDM----------------------------------------------  484
P.hodgsonii            IFIQG-----WPCVTLSV-------------LGCLYCYAHVGIAWAHTYSGN--------  493
B.mutus                IFIQG-----WPWVTLSV-------------LGFLYCYSHVGIAWAHAYSGN--------  493
C.elegans              FLIEETITFRWPFLLIIS-------------LGWFHANTAMEV--DRLKSLEKSKKEKSN  519
                       ::::
```

FIGURE S1. Multiple sequence alignment of HHAT sequences from 22 species. Clustal Omega sequence alignment of HHAT sequences from 22 different species. Cysteines are highlighted in yellow except for Cys324 which is highlighted in green. The critical MBOAT residues Asp339 and His379 (numbering from human sequence) are highlighted in red. Sequences aligned: *H. sapiens* [NP_001116306.1*], M. musculus* [NP_659130.2], *M. fascicularis* [XP_005540793.1], *S. scrofa* [XP_005667927.1], *P. troglodytes* [JAA02252.1], *P. paniscus* [XP_003814251.1|], *C. hircus* [XP_005691086.1], *C. lupus familiaris* [XP_005622394.1], *N. leucogenys* [XP_003273071.1], *O. orca* [XP_004275337.1], *C. sinum sinum* [XP_004424926.1], *D. rerio* [XP_005158569.1], *C. elegans* [NP_001022489.1], *R. norvegicus* [XP_002724948.2], *A. mississippiensis* [XP_006266096.1], *T. chinensis* [XP_006159401.1], *V. pacos* [XP_006198798.1], *C. ferus* [XP_006180810.1], *P. sinensis* [XP_006136963.1*], M. lucifugus* [XP_006089978.1], *L. chalumnae* [XP_006007432.1], *P. hodgsonii* [XP_005981119.1], *B. mutus* [XP_005888635.1].
